# CRISPR/Cas9 mediated gene correction ameliorates abnormal phenotypes in spinocerebellar ataxia type 3 patient-derived induced pluripotent stem cells

**DOI:** 10.1038/s41398-021-01605-2

**Published:** 2021-09-17

**Authors:** Lang He, Shang Wang, Linliu Peng, Huifang Zhao, Shuai Li, Xiaobo Han, Jean de Dieu Habimana, Zhao Chen, Chunrong Wang, Yun Peng, Huirong Peng, Yue Xie, Lijing Lei, Qi Deng, Linlin Wan, Na Wan, Hongyu Yuan, Yiqing Gong, Guangdong Zou, Zhiyuan Li, Beisha Tang, Hong Jiang

**Affiliations:** 1 Department of Neurology, Xiangya Hospital, Central South University, Changsha, Hunan China; 2 School of Life Sciences, University of Science and Technology of China, Hefei, China; 3 University of Chinese Academy of Sciences, Beijing, China; 4 Key Laboratory of Hunan Province in Neurodegenerative Disorders, Central South University, Changsha, Hunan China; 5 National Clinical Research Center for Geriatric Diseases, Central South University, Changsha, Hunan China; 6 Department of Pathology, Xiangya Hospital, Central South University, Changsha, Hunan China; 7 CAS Key Laboratory of Regenerative Biology, Guangdong Provincial Key Laboratory of Stem Cell and Regenerative Medicine, Guangzhou Institutes of Biomedicine and Health, Chinese Academy of Sciences, Guangzhou, China; 8 GZMU-GIBH Joint School of Life Sciences, Guangzhou Medical University, Guangzhou, China; 9 Department of Anatomy and Neurobiology, Xiangya School of Medicine, Central South. University, Changsha, Hunan China; 10 Laboratory of Medical Genetics, Central South University, Changsha, Hunan China; 11 School of Basic Medical Science, Central South University, Changsha, Hunan China; 12 Hunan International Scientific and Technological Cooperation Base of Neurodegenerative and Neurogenetic Diseases, Changsha, China

**Keywords:** Stem cells, Molecular neuroscience, Personalized medicine

## Abstract

Spinocerebellar ataxia type 3/Machado–Joseph disease (SCA3/MJD) is a progressive autosomal dominant neurodegenerative disease caused by abnormal CAG repeats in the exon 10 of *ATXN3*. The accumulation of the mutant ataxin-3 proteins carrying expanded polyglutamine (polyQ) leads to selective degeneration of neurons. Since the pathogenesis of SCA3 has not been fully elucidated, and no effective therapies have been identified, it is crucial to investigate the pathogenesis and seek new therapeutic strategies of SCA3. Induced pluripotent stem cells (iPSCs) can be used as the ideal cell model for the molecular pathogenesis of polyQ diseases. Abnormal CAG expansions mediated by CRISPR/Cas9 genome engineering technologies have shown promising potential for the treatment of polyQ diseases, including SCA3. In this study, SCA3-iPSCs can be corrected by the replacement of the abnormal CAG expansions (74 CAG) with normal repeats (17 CAG) using CRISPR/Cas9-mediated homologous recombination (HR) strategy. Besides, corrected SCA3-iPSCs retained pluripotent and normal karyotype, which can be differentiated into a neural stem cell (NSCs) and neuronal cells, and maintained electrophysiological characteristics. The expression of differentiation markers and electrophysiological characteristics were similar among the neuronal differentiation from normal control iPSCs (Ctrl-iPSCs), SCA3-iPSCs, and isogenic control SCA3-iPSCs. Furthermore, this study proved that the phenotypic abnormalities in SCA3 neurons, including aggregated IC2-polyQ protein, decreased mitochondrial membrane potential (MMP) and glutathione expressions, increased reactive oxygen species (ROS), intracellular Ca^2+^ concentrations, and lipid peroxidase malondialdehyde (MDA) levels, all were rescued in the corrected SCA3-NCs. For the first time, this study demonstrated the feasibility of CRISPR/Cas9-mediated HR strategy to precisely repair SCA3-iPSCs, and reverse the corresponding abnormal disease phenotypes. In addition, the importance of genetic control using CRISPR/Cas9-mediated iPSCs for disease modeling. Our work may contribute to providing a potential ideal model for molecular mechanism research and autologous stem cell therapy of SCA3 or other polyQ diseases, and offer a good gene therapy strategy for future treatment.

## Introduction

Spinocerebellar ataxia type 3/Machado–Joseph disease (SCA3/MJD) is the most common subtype of spinocerebellar ataxias (SCAs), accounting for about 60–70% of SCAs in the Chinese population [1–3]. The pathogenesis of SCA3 is caused by the abnormal CAG repeats in the encoding region of *ATXN3*. The normal CAG repeats expand from 11 to 44, and when the dynamic expansions up to 60–87, a related disease is diagnosed [4–6]. Abnormally CAG expansions result in abnormal polyglutamine (polyQ) tract in the encoded ataxin-3 protein, forming neuronal intranuclear inclusions (NIIs) selectively accumulated in the specific regions of the nervous system (cerebral cortex, cerebellum, brain stem and spinal cord, etc.) [7]. This disease is one of the representative diseases of polyQ diseases. Previous studies on neuropathology have been conducted in animal models of polyQ diseases, but they cannot fully simulate all aspects of human neuronal degeneration. Therefore, it is very important to select a disease model that effectively simulates neuronopathy. human-induced pluripotent stem cells (hiPSCs) carry the entire genetic background of patients, which have the potential to proliferate and differentiate into cells of interest indefinitely [7–10]. iPSCs can be used as an ideal source of cells for neuronal damage in SCA3, by inducing differentiation into specific neuron types [11].

The pathogenesis of SCA3 is complex, among which mitochondrial dysfunction and oxidative stress disorders are the focus of research on polyQ diseases [12–14]. In polyQ diseases, studies have found that when mitochondrial dysfunction occurs, mitochondrial membrane structure and permeability changes, cellular Ca^2+^ influx increases, Ca^2+^ homeostasis disorders, and mitochondrial membrane potential (MMP) decreases [14–16]. When oxidative stress disorder occurs, antioxidant enzymes such as glutathione (GSH) decrease, reactive oxygen species (ROS) production increases, and malondialdehyde (MDA) increases due to lipid peroxidation [17–19]. These results further suggest that mitochondrial dysfunction and oxidative stress disorders play a key role in the neuronal dysfunction in polyQ diseases, but the specific mechanism remains unclear. Therefore, further investigation of mitochondrial dysfunction and oxidative stress disorders in SCA3-iPSCs will lay an important experimental foundation for the development of targeted treatment strategies based on phenotypes.

Gene therapy provides an important means for repairing pathogenic genes of polyQ diseases. RNA interference (RNAi) and antisense oligonucleotide (ASO) are potentially effective strategies for the treatment of polyQ diseases via inhibiting gene expression by targeting the mRNA, either deleting CAG expansions via the selective method, or silencing wild type *ATXN3 or HTT* via non-selective method [20, 21]. However, polyQ-related genes (both wild type and mutant alleles) were mainly inhibited by the RNAi and ASO, which are linked with some problems such as non-specific, high off-targeted sites (OTs), easy to degrade over time, and the curative effect is not long-lasting. Besides, these gene silencing technologies cannot accurately repair mutant gene [20–24]. Therefore, it is of far-reaching significance to develop gene-editing therapy strategies that are specific and heritable for permanent silencing or even repairing DNA sequences. Zinc finger nucleases (ZFNs) and transcription activator-like effector nucleases (TALENs) are the earliest permanent gene-editing techniques [25]. Compared with ZFNs and TALENs, the clustered regularly interspaced short palindromic repeats (CRISPR/Cas9) has the advantages of high mutation induction rate, low cost, ease of customization, and multiplex genome editing [26], it has become a reliable molecular operating system in that repair abnormal CAG repeats.

CRISPR/Cas9 consists of small guided RNA nucleases (sgRNAs) and Cas9 protein, containing 20nt nucleotide sequences complementary to the target DNA, which can specifically target the 5-NGG′ protospacer adjacent motif (PAM), and silence targeted gene permanently. Double-strand breaks can be repaired by non-homologous end-joining (NHEJ) or homologous recombination (HR). NHEJ repair results in frameshift mutation in open-reading frames, premature translation termination, and transcript degradation mediated by nonsense translation. However, HR can carry out accurate and specific repair at the gene target sites requiring the exogenous introduction of a repair template, thus avoiding the above problems caused by NHEJ repair [26–28]. At present, there is only one CRISPR/Cas9 genome-editing therapy for SCA3-iPSCs, and it mainly relies on NHEJ to delete the abnormally CAG expansions to form truncated ataxin-3 protein [29]. However, it is not clear whether this truncated protein can fully exert its normal protein function and produce non-toxic variants in vivo and in vitro. Previously, An et al. and Xu et al. used HR strategy to accurately repair abnormal CAG expansions in the exon 1 of the *HTT* gene, which significantly improved the phenotypic abnormalities [10, 30]. Therefore, the gene targeting technology based on HR is a feasible and targeted way of precise gene modification, which shows great application prospects and advantages in the treatment of hereditary diseases such as HD. Additionally, the combination of Cas9 nickase (Cas9n) and paired sgRNAs can achieve effective cleavage of the target site and reduce the occurrence of OTs [28, 31]. Therefore, in this study, CRISPR/Cas9 genome-editing strategies of paired sgRNAs/Cas9n and HR targeting target gene loci will be used to accurately repair SCA3 patient-specific iPSCs.

Studies have shown that CRISPR/Cas9 genome-editing technology can effectively improve the phenotype of diseases, such as DMD, ALS, and HD [32–34]. In HD-induced iPSCs, CRISPR/Cas9 genome-editing technology can improve neuronal cell death, mitochondrial oxidative phosphorylation level, ATP production, and mitochondrial oxygen consumption phenotypes [10, 30]. However, the application of CRISPR/Cas9 genome-editing technology in the treatment of SCA3 disease phenotype is rarely studied, and whether this technology can play a therapeutic role in SCA3 urgently needs further study. Previous studies have obtained two strains of iPSCs derived from urothelial cells of SCA3 patients (CAG repeats is 31/74 and 26/76, respectively). SCA3/MJD-iPSCs own the same mutant *ATXN3* as parental somatic cells and have the potential to differentiate into neural stem cells (NSCs) and neuronal cells, which provides a good cell model for the study of pathological mechanisms and drug screening of SCA3 [35, 36]. In this study, we firstly use the strategy of paired sgRNAs/Cas9n and HR to precisely repair the abnormal CAG expansions in the *ATXN3* of SCA3-iPSCs. Meanwhile, we investigate the abnormal phenotypes such as mitochondrial function and oxidative stress levels in the corresponding cerebral cortical neurons, and isogenic control SCA3-iPSCs investigate the relevant molecular phenotypes under the genetic backgrounds. In summary, our study sets the crucial stage for autologous stem cell gene therapy of polyQ diseases such as SCA3 in vitro and provides a landmark and effective method to overcome the difficult problem of these diseases.

## Materials and methods

### Cloning of sgRNAs and donor DNA

Two guide sgRNAs (Table S1) were designed to target the *ATXN3* using CRISPR design tool (E-CRISP: http://www.e-crisp.org/E-CRISP/) [37], and cloned into the spCas9 plasmids pX330-mcherry from Feng Zhang (Addgene #98750), adopted from Ran et al. [26]. A 1.9 kb 5′homology arm and a 3.2 kb 3′homology arm containing 17 “CAG” repeat in the exon 10 of *ATXN3*, were cloned into the HindIII and NotI sites (Life Technologies, USA) of the pFlexible-DT targeting donor vector (loxP-pGK-puro-loxP).

### The activity of sgRNAs/Cas9n in HEK293T cells

HEK293T cells were cultured in high glucose DMEM medium (Gibco, USA) supplemented with 10% fetal bovine serum (Life Technologies, USA), 3 × 10^5^ HEK293T cells were seeded six-well plates and transfected with 3 µg DNA using Effectene^®^ Transfection Reagent (QIAGEN, Germany) according to the manufacturer’s instructions. For testing the activity of sgRNAs/Cas9n, after 48 h transfection, the cells were lysed with NP40, targeted CAG repeats with P5–P6 primers by PCR amplification and PCR products were purified, then digested with T7EN1 assay after denaturing and annealing reaction, and identified with 2% agarose gel. The percent of sgRNAs/Cas9n cutting rates were performed using ImageJ software.

### Nucleofection of sgRNA/Cas9n into iPSCs and positive clones screening

iPSCs (SCA3-001-iPSCs, CAG repeats is 31/74) were used in CRISPR/Cas9 genome-editing technology [35, 36]. Amaxa nucleofection system (Lonza, Switzerland) of iPSCs was performed following the manufacturer’s instructions. Briefly, hiPSCs were pre-treated with 10 µM ROCK inhibitor Y-27632 (Selleck, USA) for 24 h and dissociated into single-cell suspension with accutase (Sigma, USA). 6–8 × 10^5^ hiPSCs were plated in an electroporated cuvette using solution II 82 µL supplemented with the solution I 18 µL (Lonza, Switzerland). The cells were electro-transfected with 3 µg sgRNA1 and sgRNA2, 10 µg donor plasmids using program A-023 (Lonza, Switzerland). After electro-transfection, cells were immediately plated in mTeSR1 (Stem Cell Technologies, Canada) with Y-27632 for 72 h culture, and 0.3 µg/ml Puromycin (Puro) was added to the medium. After 10–14 days of selection, visible colonies were picked manually and transferred into 48-well plates, then the positive clones were screened using P1–P2, P3–P4, and *ATXN3*-FAM primers (Table S2) performed with 2 × Taq Plus Master Mix II (Vazyme, China). PCR products were run on a 2% gel-electrophoresis and further analyzed by capillary electrophoresis. In addition, the bands were purified and cloned into Ta vectors and analyzed by Sanger sequencing of Beijing TsingKe Biotechnology Company, China.

### OTs analysis

We used the GT-Scan website (https://gt-scan.csiro.au/gt-scan) [38] to detect potential OTs, potential sgRNAs OTs in the human genome using the following criteria: NRG-PAM, 3–4 mismatches. Primers were designed according to the potential 5 OTs of each sgRNAs (Table S3). The non-specific cutting of potential sgRNAs was verified by T7EN1 (NEB, USA) assay following the manufacturer’s instructions

### Whole-genome sequencing

Whole-exome sequencing of the parental CAG74 hiPSCs and two isogenic control lines (SCA3-C3 and SCA3-C12) were performed. Single nucleotide polymorphism (SNP), copy number variation (CNV), and potential sgRNAs off-target loci were analyzed among the groups. As is shown in Supplemental Experimental Procedures for the detailed protocols.

### iPSCs culture and differentiation

iPSCs were reprogrammed from urothelial cells with the non-integration method (oriP/EBNA1-based episomal vector *pCEP4*-*O2SET2K* carrying the *OCT4* (*POU5F1*), *SOX2*, *SV40LT*, *KLF4,* and *pCEP4-miR302-367* cluster) as previously described [35, 39]. iPSCs were grown on Matrigel (Biocoat, China) coated plate, cultured daily with mTeSR1 medium, and passaged with 0.5 mM EDTA (Life Technologies, USA) every 3–5 days.

According to the protocols for terminal cerebellar cortical differentiation [40], iPSCs were confluent to 90–100%, then the medium was switched to N2B27 + 2i differentiation medium [DMEM/F12 or Neurobasal medium (mixture rate: 1:1) with 1% N2, 2% B27, 1% non-essential amino acids (NEAA), 1% GlutaMax (all from Life Technologies, USA), 100 µM β-mercaptoethanol (Sigma, USA), 2 μg/mL heparin (Sigma, USA), 5 μg/mL insulin (Sigma, USA), 1 µM Dorsomorphin (Sigma, USA) and 10 µM SB431542 (Selleck, China)]. The medium was refreshed every day until 8 days of culture. The neuroepithelial aggregates were picked into the Matrigel-coated six-well plate for further differentiation in N2B27 medium without 2i, and the medium was changed daily. Around 14–18 days, the neural rosette structures appeared, the cells were suspended to expand the cultured neural stem cells (NSCs) in the N2B27 medium. After expansion 1–3 generations, the NSCs aggregates enzymatically dissociated to single cells using accutase and cultured in N2B27 medium for final neuronal differentiation.

### RNA extraction and quantitative RT-qPCR

Total RNA samples were isolated with Trizol reagent (Invitrogen, USA) and cDNA was prepared using 5 × HiScript II qRT SuperMix IIa (Vazyme, China) according to the manufacturer’s instructions. Real-time Quantitative PCR was performed with 2 × RealStar Power SYBR Mixture (GenStar, China) and curried out on the CFX96™ Real-time PCR (Bio-Rad, USA). CT-values were normalized to the GADPH using the 2^−ΔΔCT^ method. The primers were listed in Table S4.

### Immunocytochemistry

Cells were fixed with 4% paraformaldehyde for 15 min at room temperature (RT). Then, the cells were permeabilized with 0.5% Triton X-100 (Sigma, USA) for 5 min and blocked with 10% goat serum (Millipore, USA) diluted in 0.5% Triton X-100 and PBS for 30 min at RT. Primary antibodies were diluted into blocking liquid and incubated overnight at 4 °C. Then, cells were transferred into secondary antibodies and incubated for 1 h at RT in dark. Primary and secondary antibodies were listed in Table S5. The nucleus was stained with DAPI (Beyotime, China) for 5 min. Coverslips were mounted with a fluorescence quencher. Images were captured with IX73 Olympus inverted microscope (Olympus, Japan) or confocal microscope imager LSM710 or LSM800 (Zeiss, Germany), and image analyses were performed with software ZEN (Zeiss, Germany). Neuronal markers of TUJ1, MAP2, GABA, GFAP, SYP1, and PSD95, were measured by the fluorescence quantitative analysis using ImageJ software. 5–8 images were analyzed from each image.

### Mitochondrial membrane potential detection

Two strains of SCA3 patient-derived iPSCs (SCA3-001-NCs and SCA3-002-NCs) were used to detect the mitochondrial function and oxidative stress-related indicators. The cell culture medium was removed, detached cells were collected and washed with PBS 2–3 times. Then, the cells were added into JC-1 staining working solution and incubated for 30 min at 37 °C. After incubation, the cells were washed two times with JC-1 staining buffer and performed with Flow cytometry (BD Accuri C6, USA). Data were processed with Flow Jo software.

### ROS, Ca^2+^, MDA and GSH levels

The ROS was measured with DCFH-DA fluorescent probe, the intracellular Ca^2+^ were detected by the Fluo-4 AM, the MDA levels and GSH levels were detected by the Lipid Peroxidation MDA Assay Kit and reduced glutathione/oxidized glutathione (GSH and GSSG Assay Kit) following the manufacturer’s instructions (Beyotime, China), respectively. The detailed procedures are described in Supplementary Experimental Procedures.

### Electrophysiology

Whole-cell voltage-clamp and current-clamp techniques were used to conduct electrophysiological experiments at RT according to previous protocols [41]. Neurons were grown on cover glass, which was placed in the center of the cell perfusion tank. The cells were immersed in the artificially configured extracellular fluid, containing 95% O_2_ and 5% CO_2_. The resistance value of glass microelectrode used in the operation was about 8–10 MΩ. Extracellular fluid consisted of artificially simulated cerebrospinal fluid, including NaCl 127 mM, KCl 3 mM, MgSO_4_ 1 mM, NaHCO_3_ 26 mM, NaH_2_PO_4_ 1.25 mM, CaCl_2_ 2 mM, and d-glutacose 10 mM, pH = 7.3–7.4. Intracellular solutions composed of potassium-methyl sulfonate 140 mM, NaCl 5 mM, CaCl_2_ 1 mM, HEPES 10 mM, EGTA 0.2 mM, ATPNa_2_ 3 mM, and GTPNa_2_ 0.4 mM, pH = 7.2. In the current clamping mode, by recording and measuring the resting membrane potentials and spontaneous firing rate, the average value was 60 s in the gap-free mode. Then, the resting membrane potential of each cell was clamped to −70 mV as far as possible with steady currents. Action potentials threshold were recorded by depolarizing current steps (5 pA, 20 ms). Voltage-dependent ion channels recording: series resistance was compensated to about 90% before recording, the inward and outward currents were recorded. Action potential and current properties were analyzed using Clampfit 10.2 software. For postsynaptic potential recording: the spontaneous glutamatergic excitatory postsynaptic potential (EPSC) was detected at −65 mV clamping voltage. Moreover, the inhibitory postsynaptic potential (IPSC) was detected at 0 mV clamping voltage, and the data were analyzed using Clampfit 10.2 and Origin8.6 software.

### Statistical analysis

The data were analyzed with GraphPad Prism 8.0. The double-tailed *t* test was used for two groups; multi-group data were analyzed using one-way ANOVA with Bonferroni post-hoc test. All the samples were analyzed in triplicate or more independent experiments. The difference of *P* < 0.05 was statistically significant.

## Results

### Analysis of paired *ATXN3*-sgRNAs/Cas9n activity in HEK293T cells

**Fig. 1:**
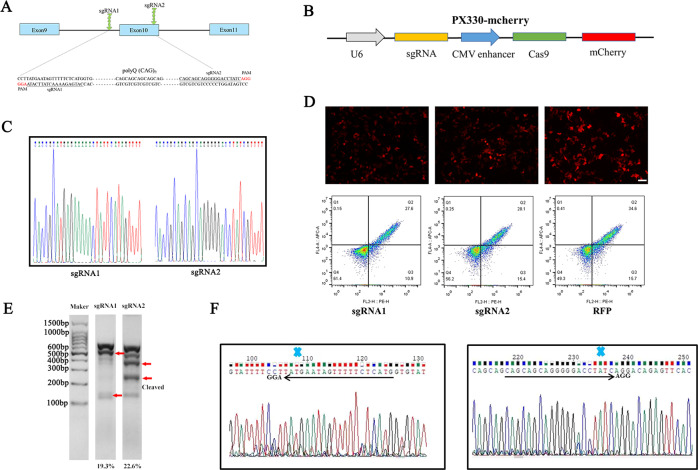
Analysis of ATXN3-sgRNAs/Cas9n cleavage activity in HEK293T cells. **a** SgRNA1 and sgRNA2 were designed to target CAG repeats located in the exon 10 of the *ATXN3* NGG PAM sequences are highlighted in red. **b** Illustration depicting the CRISPR/Cas9 expression plasmid (PX330) co-express Cas9 protein with the mCherry reporter marker and sgRNA under the U6 promoter. **c** Sequence of sgRNA1 and sgRNA2 successfully cloned into PX330 plasmid. **d** The transfection efficiency of paired sgRNAs transfected into HEK293T cells were detected by immunofluorescence and flow cytometry, the transfection efficiency of sgRNA1, sgRNA2, and RFP was 38.5%, 43.5%, and 50.3%, respectively. Scale bar: 100 μm. **e** Analysis of sgRNA1 and sgRNA cleavage activity in HEK293T cells by T7EN1 assay. The length of the targeting PCR product is 630 bp (uncleaved band). Cleaved bands were marked with red arrow, sgRNA1 and sgRNA2 cleavage efficiency reached 19.3% and 22.6%, respectively. **f** After genome-editing with CRISPR/Cas9, the sequence trace after the break site comprised a mixture of signals derived from the unmodified and modified DNA.

Paired sgRNAs can enhance Cas9-mediated double-strand breaks to generate highly specific genome editing, which could reduce OTs and enhance HR repair [28]. Considering the distance and cleavage length of sgRNAs at the target gene, we designed a pair of sgRNAs (*ATXN*3-sgRNA1 and *ATXN3*-sgRNA2) for the upstream and downstream PAM regions of CAG repeats in the exon 10 of *ATXN3* (Fig. 1a). Cells were transfected with PX330 plasmids expressing both wtCas9 protein and sgRNA (Fig. 1b). The sgRNA1 and sgRNA2 targeting CAG repeats of *ATXN3* have been successfully constructed by Sanger sequencing (Fig. 1c). The first screening of sgRNAs activity was performed in HEK293T cells. The transfection efficiency of sgRNA1, sgRNA2, and RFP was 38.5%, 43.5%, and 50.3%, respectively, by immunofluorescence and flow cytometry after 24–48 h transfection (Fig. 1d). The cellular genome was isolated after 48–72 h transfection. PCR amplification of the CAG repeats in *ATXN3*, and subsequent T7EN1 assay resulted in multiple bands in both treated and untreated control cells, where 630 amplified bands were produced in untreated cells. 116 and 514 bp bands appeared after sgRNA1 cleavage, while 253 and 377 bp bands were presented after sgRNA2 cleavage. The cleavage efficiency of sgRNA1 and sgRNA2 was 19.3% and 22.6%, respectively (Fig. 1e). HEK293T cells contained 14 and 24 CAG duplicates in both alleles of *ATXN3* (Fig. S1). After the treatment of sgRNA1 and sgRNA2, the PCR amplification of targeting site and sequencing analysis showed bimodal and mixed signals (Fig. 1f). These results indicated that the designed sgRNAs have significant cleavage efficiency in vitro experiments.

### Gene correction of SCA3 patient-derived iPSCs

**Fig. 2:**
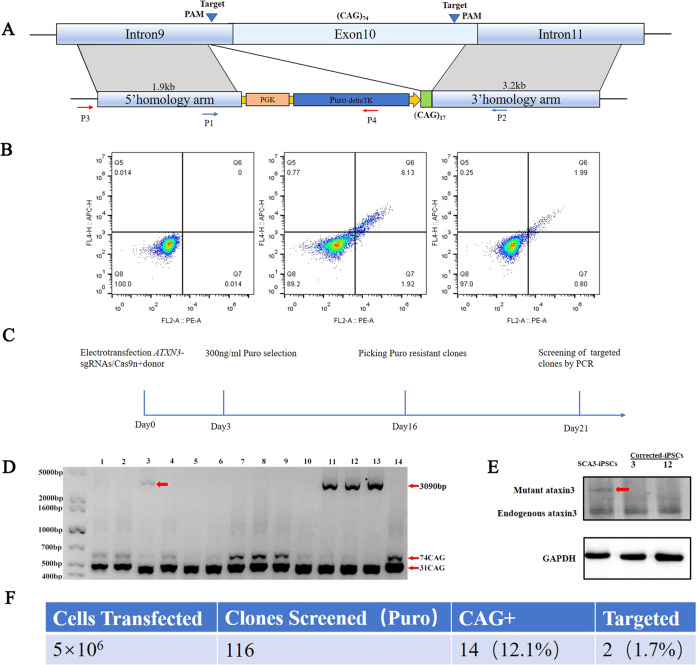
Gene correction of SCA3-iPSCs using CRISPR/Cas9 system. **a** Schematic depicting the donor vector and Cre-loxP-based HR selection strategy used for targeting the *ATXN3* locus, the targeting vector (pFlexible-DT) with left and right arms of 1.9 and 3.2 kb, respectively, and the 17 CAG repeats and Puro cassettes inserted upstream of the corrected region. The targeted fragment sizes were verified by P1–P2 and P3–P4 primers. **b** Quantification of the transfection efficiency of *ATXN3*-targeted sgRNAs, detected by flow cytometry, negative cell population (the left figure), 10% RFP positive cells (the middle figure), and 2.8% paired sgRNAs/Cas9n (sgRNA1 + sgRNA2) positive cells (the right figure). **c** Overview of the targeting workflow, including electroporation, selecting, and screening. **d** PCR-based screening for successfully targeted SCA3-iPSCs, the positive clones (C3, C11, C12, C13) contained 510 bp (31 CAG) and 3090 bp using P1–P2 primers, while the mutant bands (639 bp) indicated the 74 CAG repeats shown in red arrow. **e** Verification of successful correction at the *ATXN3* locus in corrected SCA3-iPSCs by Western blotting, the C3 and C12 clones indicated no expanded polyQ tracts, the red arrow indicates the mutant ataxin-3 protein. **f** Summary of targeted efficiency of CRISPR/Cas9 genome editing HR was 1.7%.

To correct the disease mutation in SCA3-iPSCs and generate isogenic control lines, we adopted the CRISPR/Cas9 and Cre-loxP-mediated HR-based genome editing methods. We employed the targeting donor construct (pflexible-DT vector), the donor cassette having 1919 bp left arms and 3264 bp right arms containing 17 CAG repeats based on the sgRNA1 cleavage site (intron 9). It was successfully cloned into a pFlexible-DT vector, and Puro-resistance gene and PCR (P1–P2 and P3–P4) for positive clone screening (Fig. 2a). Firstly, we transfected 3 μg paired sgRNAs/Cas9n (sgRNA1 + sgRNA2) and 10 μg donor repair template into the SCA3-iPSCs carrying 31/74 CAG expansions. The paired sgRNAs/Cas9n (sgRNA1 + sgRNA2) transfecting efficiency was 2.8% by flow cytometry (Fig. 2b). After post-electroporation for 72 h, 300 ng/ml Puro was added for selecting corrected clones and targeted clones were selected for further culture on 10–18 days (Fig. 2c). Positive clones were identified by PCR using P1–P2 primers. The successfully corrected cell lines contained 510 and 3090 bp and identified four corrected lines (C3, C11, C12, C13). Moreover, corrected lines did not detect any mutant bands (Fig. 2d). In addition, the positive clones were further verified by PCR (P3–P4 primers), and only two cell lines (C3, C12) contained 3787 bp target bands (Fig. S2). We confirmed that paired sgRNAs effectively targeted CAG expansions in the exon 10 of *ATXN3*.

Successful correction of the mutant *ATXN3* allele was verified by Western blot using an ataxin-3 antibody (H9, MAB5360) (Fig. 2e). Of the 116 clones screened, 14 were targeted for CAG expansions, of which 2 clones (C3 and C12) were confirmed by PCR screening (P1–P2 and P3–P4 primers) and Western blot, accounting for about 1.7% of the screened positive clones and the HR rate was found to be consistent with previously reported finding in HD-iPSCs [30] (Fig. 2f). Capillary electrophoresis and fragment length analysis showed that the corrected SCA3-iPSCs (SCA3-C3 and SCA3-C12) did not contain visible disease-causing *ATXN3* mutations (74 CAGs). Meanwhile, the isogenic control SCA3-iPSCs maintained 17/31 CAG repeats in *ATXN3* (Fig. S3).

Remarkably, ten potential OTs for *ATXN3*-sgRNAs/Cas9n were predicted by silico analysis. The potential OTs of each sgRNAs were detected by T7EN1 assays. Our results showed no detectable OTs examined in the potential 10 sites (Fig. S4). Corrected SCA3-iPSCs (SCA3-C3 and SCA3-C12) and parental SCA3-iPSCs were sequenced by whole-genome sequencing, the results showed that did not produce too many specific SNP sites by CRISPR/Cas9, which did not affect the gene stability after genome-editing cell lines. Furthermore, these loci were compared with sgRNA sequences, and it was found that these loci did not exist in the potential off-target region of sgRNAs. In addition, no significant CNV and genomic sequence changes were found in the genome-edited cell lines, suggesting that no OTs and interruptions appeared in isogenic control SCA3-iPSCs (SCA3-C3 and SCA3-C12) compared to parental SCA3-iPSCs, as were shown in Figs. S5, S6, and Table S6.

### Corrected SCA3-iPSCs remaining pluripotent characteristics

**Fig. 3:**
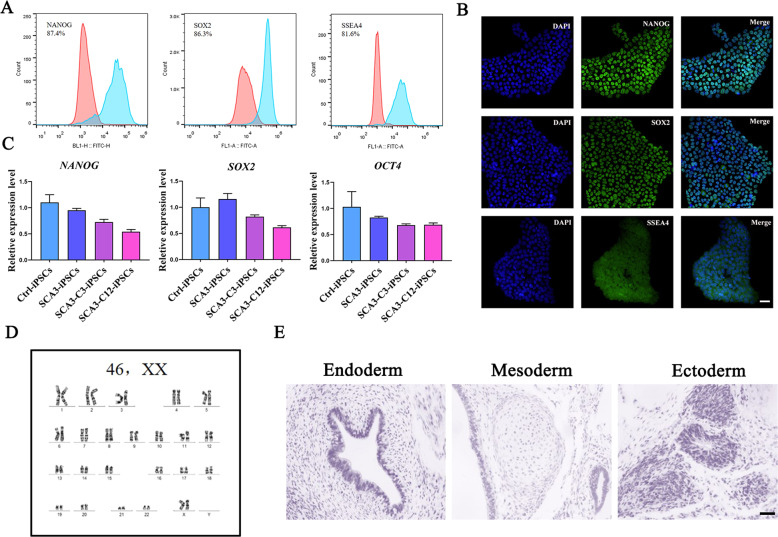
Corrected SCA3-iPSCs (SCA3-C3-iPSCs) maintained pluripotent characteristics and normal karyotype. **a** Flow cytometry of pluripotent markers, including nuclear staining for NANOG, OCT4, and cell surface staining for SSEA4. **b** Immunofluorescence staining of pluripotent markers, including nuclear staining for NANOG, SOX2, and cell surface staining for SSEA4. Scale bar: 100 μm. **c** Total RNA was isolated from iPSCs and analyzed with RT-qPCR. Primers used for *NANOG*, *OCT4*, and *SOX2* specifically detect the transcripts from the endogenous genes. **d** Karyotyping and G-band analysis showed corrected SCA3-iPSCs have a normal 46, XX karyotype. **e** Teratomas test comprised derivatives of the 3 germ layers. Scale bar: 100 µm. SCA3-iPSCs: SCA3 patient-derived iPSCs. Ctrl-iPSCs: healthy control iPSCs. SCA3-C3-iPSCs and SCA3-C12-iPSCs: corrected SCA3-iPSCs.

Previous studies showed that parental SCA3-iPSCs retained disease-associated mutations and normal karyotype, expressing pluripotency markers, and have the potential to differentiate into three germ layers [35]. The pluripotent characteristics are also kept in the genetically control SCA3-iPSCs (SCA3-C3 and SCA3-C12) (Figs. 3 and S7). Specifically, normal karyotype (Fig. 3d) and pluripotency markers of NANOG, SOX2, SSEA4 were measured by immunofluorescence staining (Fig. 3b) and flow cytometry (Fig. 3a). Endogenous expressing pluripotency markers of *NANOG*, *SOX2*, and *OCT4* were evaluated by RT-qPCR (Fig. 3c). In vivo teratoma assay, the corrected clones showed the potential to differentiate into three germ layers, as shown by positive hematoxylin dyeing for glandular structure (endoderm), cartilage (mesoderm), and neural rosettes (ectoderm) (Fig. 3e).

### Differentiation of SCA3 and isogenic control iPSCs into cerebral cortical neurons and hindbrain Purkinje progenitor cells

**Fig. 4:**
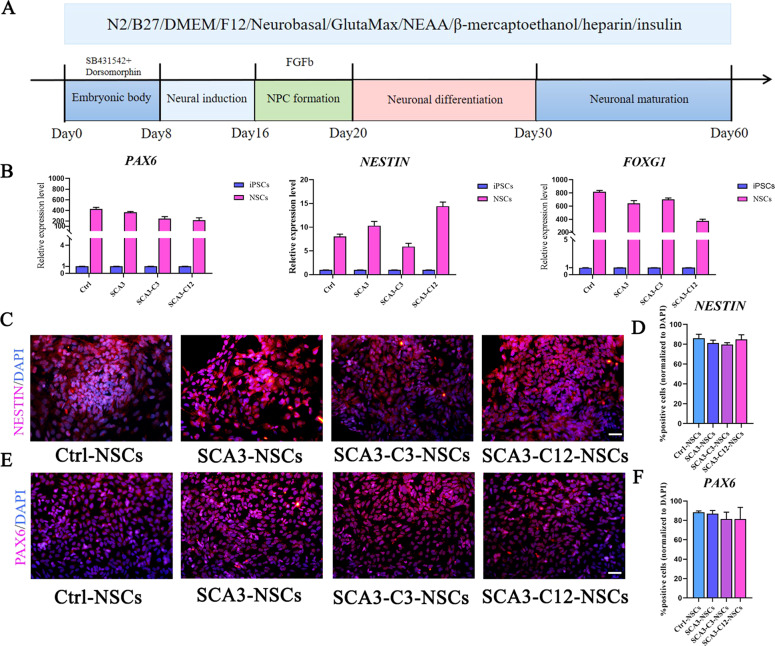
The similar differentiation markers expressing in SCA3-NSCs and isogenic control SCA3-NSCs. **a** The scheme of neuronal differentiation protocol in vitro, the mature neurons were obtained about 60 days after differentiation. **b–f** Neural induction resulted in robust expression of NSCs. **b** NSCs markers, *PAX6*, *NESTIN*, *FOXG1* (day 16) as measured by qRT-PCR (*n* = 3 per sample independent biological replicates), the higher expression of NSCs markers in NSCs stage compared with iPSCs stage. **c–f** PAX6 and NESTIN (day 16) as detected by immunocytochemistry. There was no difference in each group (*P* > 0.05). Scale bar: 100 µm. **d**, **f** All data were presented as mean ± SD. The data was determined by one-way ANOVA (*P* = 0.05) and Bonferroni post-hoc test among the groups. *n* = 5 images for immunofluorescence. Ctrl-NSCs: healthy control NSCs. SCA3-NSCs: SCA3 patient-derived NSCs. SCA3-C3-NSCs and SCA3-C12-NSCs: corrected SCA3-NSCs.

To generate mature neurons from SCA3-iPSCs, Koch et al. differentiated iPSCs into long-term self-renewing neuroepithelium stem cells. The differentiation system has cortical neural and glial mixed populations after long-term proliferation [42]. Given that studying iPSCs-derived cerebral cortical neurons may shed light on the pathogenesis of SCA3, we used the monolayer culture method of cortical neurons, which experienced the stage of neural rosettes and mature neural differentiation [40]. Typical and mature neurons can be observed after about 60 days of neural induction (Fig. 4a). SCA3-iPSCs (74 CAG repeats), corrected SCA3-iPSCs (SCA3-C3 and SCA3-C12, containing 17 and 31 CAG repeats, respectively) and healthy control iPSCs (Ctr1, 26 CAG repeats) were efficiently differentiated into NSCs after 16 days of neural induction. In the NSCs stage, RT-qPCR results showed that the NSCs surface markers (*PAX6*, *NESTIN*, *FOXG1*, *SOX1,* and *OTX1D*) were highly expressed compared with the iPSCs stage in the above groups (Figs. 4b and S8). Moreover, PAX6 and NESTIN were positive staining by immunofluorescence. At this stage, there was no significant difference in the NSCs markers expression among the groups (Fig. 4c–f). In addition, IC2-positive polyQ protein and ataxin-3 protein were expressed in the above groups. However, no polyQ aggregates were detected in the NSCs stage (Fig. S8).

**Fig. 5:**
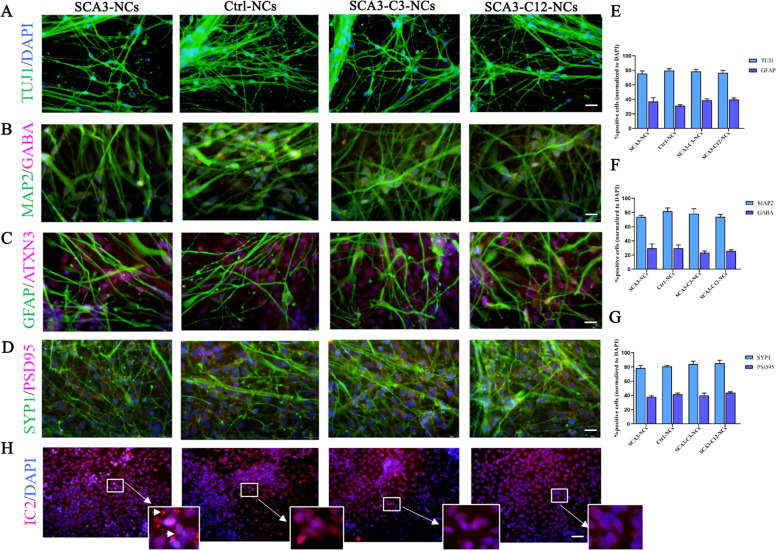
Differentiation of SCA3 and isogenic control SCA3-NSCs into cerebral cortical neurons. **a–c** Immunofluorescence was used to detect the expression of surface markers and synaptic proteins in each group. Further differentiation resulted in neurons expressing TUJ1, MAP2, GABA, and GFAP. **e** and **f** The percentage of corresponding neuron markers were counted. Similar results were acquired with each cell line in our study (*P* > 0.05). Scale bar (TUJ1): 50 µm. Scale bar (MAP2, GABA, and GFAP): 25 µm. **d**, **g** Representative images of neuronal cells stained with SYP1 and PSD95 on days 35–50, there was no difference in the expression of SYP1/PSD95 in each group (*P* > 0.05). Scale bars 25 µm. **h** IC2 positive polyQ aggregates were detected in SCA3 neurons compared with other groups on day 50 (aggregates indicated in enlarged images with the arrow). Scale bar: 100 µm. **b**, **d, f**, **h** All data were presented as mean ± SD. The data as determined by one-way ANOVA and Bonferroni post hoc test. *n* = (6, 6, 6, 6, 6, 6) images for TUJ1, MAP2, GABA, GFAP, SYP1, and PSD95 neuronal markers expression. Ctrl-NCs: healthy control neurons. SCA3-NCs: SCA3 patient-derived neurons. SCA3-C3-NCs and SCA3-C12-NCs: corrected SCA3 neurons.

Using our protocols, 30–60 days after neuronal differentiation, all cells expressed mature neuronal surface marker, cortical neurons expressed TUJ1, MAP2, and GABA positives, and astrocytes expressed glial fibrillary acidic protein (GFAP) positives. The ratio of neurons to astrocytes (TUJ1/GFAP) was about 2:1 (Fig. 5a–c). At this stage, there were no significant differences in neuronal surface marker (TUJ1, GABA, MAP2, and GFAP) among the healthy control neurons (Ctrl-NCs), SCA3 neurons (SCA3-NCs), and corrected SCA3 neurons (SCA3-C3-NCs and SCA3-C12-NCs) groups (Fig. 5e, f). Therefore, our protocols of iPSCs differentiated into neurons and astrocytes were consistent with previous studies, in which the transformation of iPSCs led to a mixture of cultured neurons and astrocytes [42, 43]. Further analysis revealed that SYP1/PSD95, which is the presynaptic marker and postsynaptic marker of synaptic development, expressed similarly in each group at days 40–60 differentiation as previously reported (Fig. 5d and g). Moreover, IC2-positive polyQ aggregates were only detected in SCA3 neurons compared with other groups, and the isogenic controls did not detect IC2-positive polyQ proteins (Fig. 5h).

Former studies have shown the susceptibility of cerebellar neurons in SCA3/MJD patients [7]. To conduct the cerebellar neurons, we adopted a specific developmental model of cerebellar tissue, by differentiating iPSCs into cerebellar Purkinje progenitor cells based on previous protocols (Fig. S9a) [44–47]. We detected the up-regulation of midbrain/hindbrain patterning markers, such as the *KIRRLE2*, *FGF8*, *WNT1*, *GBX2,* and *OTX2*, by RT-qPCR on days 24 of differentiation (Fig. S9b). Immunofluorescence showed cerebellar precursor cells expressing KIRREL2/TUJ1 on days 24–32 (Fig. S9c). After 24–30 days of differentiation, the cerebellar progenitor cells in heterogeneous culture were sorted by KIRREL2^+^, and the selected KIRREL2^+^ Purkinje progenitor cells accounted for 19.2% by flow cytometry (Fig S9d). However, the purified KIRREL2^+^ cerebellar precursor cells need to be co-cultured with purified cerebellar granule cells, which came from newborn mice, for 2–3 months until the mature Purkinje cells (PCs) generation. We could not obtain enough vigorous cell populations after fluorescence-activated cell sorting. In the future, we are planning to obtain more vigorous cell populations by optimizing the Purkinje progenitor cells purification scheme or selecting THY1^+^ cell subpopulations for further exploring mature PCs differentiation strategies [47].

### Electrophysiological functions of SCA3-iPSCs differentiated cerebral cortical neurons

**Fig. 6:**
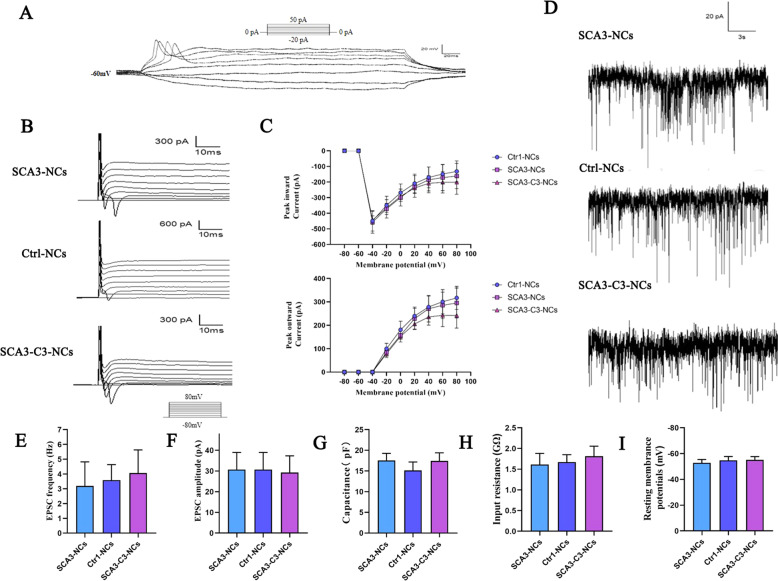
Electrophysiological characterization of cerebral cortical neurons differentiated from SCA3 and isogenic control SCA3-iPSCs. **a** Representative current-clamp recording of evoked action potentials from SCA3 neurons after injection of step currents (−20 to 50 pA). **b** Voltage clamp recordings sustained inward and outward currents, in response to depolarizing voltage steps (−80 to 80 mV) in Ctrl-NCs, SCA3-NCs, and SCA3-C3-NCs, respectively. **c** Quantification of inward and outward currents peak curves in Ctrl-NCs (*n* = 6), SCA3-NCs (*n* = 6), and SCA3-C3-NCs (*n* = 6). Similar results were detected among the groups (*P* > 0.05). **d** Representative traces of excitatory postsynaptic currents in Ctrl-NCs, SCA3-NCs, and SCA3-C3-NCs, respectively. **e** and **f** The amplitude and frequency of EPSC were similar in each group (*P* > 0.05). *n* = 7 in three groups. **g**–**i** The membrane capacitance, input resistance, and resting membrane potentials of Ctrl-NCs, SCA3-NCs, and SCA3-C3-NCs. No significant difference was shown in the three groups (*P* < 0.05). *n* = 7 in each group. All data were shown as mean ± SD. The data was determined by one-way ANOVA and Bonferroni post-hoc test. Ctrl-NCs: healthy control neurons. SCA3-NCs: SCA3 patient-derived neurons. SCA3-C3-NCs: corrected SCA3 neurons.

Next, we explored the electrophysiological properties of the differentiated neurons. Whole-cell patch-clamp recording techniques were used to measure the intrinsic electrophysiological excitability of these differentiated cells for 6–7 weeks. Evoked action potentials displaying the excitable properties under the current clamp were recorded in SCA3-NCs (Fig. 6a), which shows that neurons can produce and transmit excitation. Robustly whole-cell currents were detected in healthy control neurons (Ctrl-NCs), SCA3 neurons (SCA3-NCs), and corrected SCA3 neurons (SCA3-C3-NCs), including Na^+^, K+ inward currents, and Ca^2+^ and outward currents (Fig. 6b). Besides, the Na^+^ and Ca^2+^ currents can be blocked by TTX (1 μM) and CdCl_2_ (0.1 mM) ion antagonist, respectively (Fig. S10a). Under the action of the depolarized current pulse, there was no significant difference in the inward or outward current peaks among the three groups (Fig. 6c). In order to assess the effect of *ATXN3* mutation on postsynaptic potentials, both spontaneous glutamatergic EPSC and IPSC were recorded in whole-cell voltage-dependent recordings. Results showed that EPSC could be blocked by CNQX (AMPA receptor antagonist, 10 μM), or MK801 (NMDA receptor antagonist, 10 μM), respectively (Figs. 6d and S10b). Altogether, these data indicated that glutamate-mediated EPSC and GABA mediated IPSC input onto neuronal cells, a study supported by previous studies [42]. The frequency and amplitude of EPSC were very similar in each group (Fig. 6e, f). In addition, the membrane capacitances, input resistance, and resting membrane potentials also showed no significant difference among the three groups (Fig. 6g–i). These findings suggested that TUJ1/MAP2-positive neurons exhibit electrophysiological properties, and there was no obvious difference among the different neuronal groups.

### Reversal of mitochondrial dysfunction and oxidative stress disorders in corrected SCA3 neurons

**Fig. 7:**
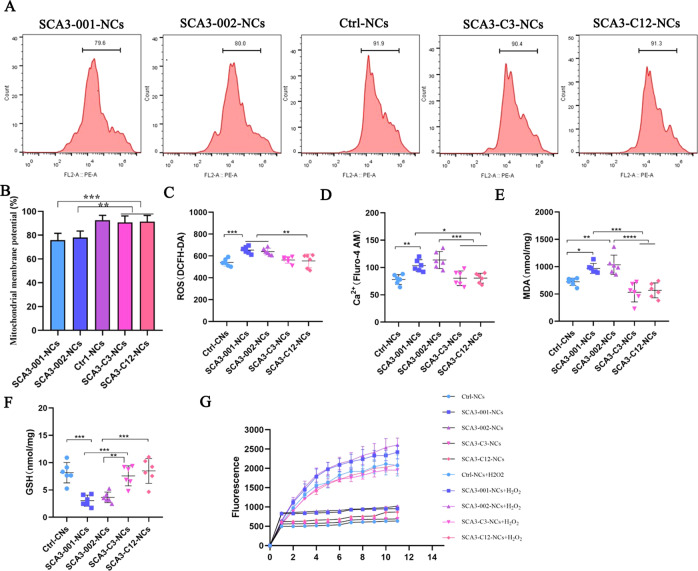
The mitochondrial dysfunction and oxidative stress phenotypic characteristics rescued in isogenic control SCA3-NCs. **a** and **b** Mitochondrial membrane potentials were decreased in SCA3 neurons (SCA3-001-NCs and SCA3-002-NCs) compared with Ctrl-NCs, SCA3-C3-NCs, and SCA3-C12-NCs (*P* < 0.05). Six independent biological replicates samples in each group. **c** Expression of ROS in each group. The ROS was increased in SCA3 neurons compared with another group, corrected neurons rescued the ROS (*P* < 0.05). **d** Ca^2+^ concentration in Ctrl-NCs, SCA3 neurons, and isogenic control cell lines. The results indicated the Ca^2+^ was increased in SCA3 neurons, the Ca^2+^ concentration was rescued in corrected neurons (*P* < 0.05). **e** and **f** MDA and GSH quantification across the above groups. The MDA levels were increased and GSH levels were reduced in SCA3 neurons compared with other groups, the MDA and GSH concentrations were rescued in the corrected neurons (*P* < 0.05). **g** ROS levels and H_2_O_2_ induced ROS were recorded every 10 min for a total of 10 times. The findings showed the ROS activated in SCA3 neurons, the disease phenotypes were rescued in the corrected neurons (*P* < 0.05). Data were shown as mean ± SD (*n* = 6). The data was calculated using one-way ANOVA, followed by Bonferroni post-hoc test. *P < 0.05, **P < 0.01, ***P < 0.005, ****P < 0.001. Ctrl-NCs: healthy control neurons. SCA3-001-NCs and SCA3-002-NCs: SCA3 neurons. SCA3-C3-NCs: corrected SCA3 neurons.

Here, we detect the phenotypes changes in two strains of SCA3-iPSCs (SCA3-001-iPSCs and SCA3-002-iPSCs) were reported in our recent study [35, 36]. In this study, MMP was decreased significantly in SCA3 neurons (SCA3-001-NCs and SCA3-002-NCs) compared with healthy control neurons (Ctr1-NCs), MMP was increased significantly in corrected SCA3 neurons (SCA3-C3-NCs and SCA3-C12-NCs) compared with SCA3 neurons (Fig. 7a, b). In the present study, the ROS and Ca^2+^ levels were significantly higher in SCA3 neurons compared with Ctr1-NCs, while obviously alleviated the changes in the corrected SCA3 neurons (Fig. 7c, d). In addition, the expression of MDA increased, and GSH decreased in SCA3 neurons but could be rescued in the corrected SCA3 neurons (Fig. 7e, f). In order to test acute treatment of sgRNA/Cas9n could alleviate the phenotypes associated with SCA3-iPSCs/neurons. We have electro-transfect the sgRNAs/Cas9 vectors into the SCA3-iPSCs/neurons, but we could not detect any phenotypes alleviation in the mixed cell lines (Fig. S11), considering the collection of acute electroporation cells, the cell viability and gene targeting efficiency is too low to display phenotypic changes. These findings indicated that the increased generation of oxygen free radicals, oxidative stress disorders, and decreased antioxidant capacity in SCA3 neurons, were related to neuronal dysfunction [17, 48]. Moreover, ROS were activated in SCA3 neurons over time following H_2_O_2_ stimulation compared with Ctrl-NCs and isogenic control SCA3 neurons (Fig. 7g). Altogether, this study highlighted the likelihood contribution of mitochondrial dysfunction and oxidative stress disorders to the pathogenesis of SCA3.

## Discussion

This study confirmed that paired sgRNAs/Cas9n and HR strategy can successfully repair the 74 CAG expansions in the exon 10 of *ATXN3*, and can effectively silence the expression of mutant ataxin-3 protein specifically. Moreover, the safety of paired sgRNAs/Cas9n strategy was detected by T7EN1 assay and whole-genome sequencing, and no potential OTs were detected, which confirmed the safety and specificity of paired sgRNAs/Cas9n strategy in SCA3-iPSCs. We further demonstrated that a series of abnormal phenotypes in SCA3-NCs, including IC2-polyQ aggregations, decreased the levels of MMP and GSH, H_2_O_2_-induced oxidative stress activation, and significantly increased ROS, Ca^2+^, and MDA levels, above phenomenons all are rescued in isogenic control SCA3 neurons.

So far, multiple gene therapies have been used to treat polyQ diseases such as SCA3. In SCA3 cell and animal models, the expression of mutant *ATXN3*(mATXN3) gene was inhibited by RNAi and ASO techniques, but the expression of wild-type *ATXN3*(wtATXN3) was also inhibited [49–51], with the drawbacks of low specificity, high OTs and transient inhibitory effect [49, 52–56]. CRISPR/Cas9 genome-editing technology can achieve specific knockout or knock-in of target genes, providing a specific, efficient, and sustainable method for gene targeting. Previous studies have shown that CRISPR/Cas9 mediated genome engineering technology has a significant therapeutic effect on mice and cell models of HD [10, 30, 34, 57–59]. These studies suggest that CRISPR/Cas9 genome editing is an effective treatment for polyQ disease. In the previous CRISPR/Cas9 genome-editing therapy of SCA3, the purpose of silencing m*ATXN3* was mainly achieved by deleting the mutated CAG repeats in the *ATXN3* [29]. Ouyang et al. knockout the mutant CAG expansions in the exon 10 of *ATXN3* through the CRISPR/Cas9 system, the gene repair mechanism were mainly carried out by the NHEJ method, and a stop codon appeared at the beginning of exon 11 to form a truncated ataxin-3 protein [29]. Although this protein retained its ubiquitinatitin-binding capacity, this study did not explore whether it plays a normal protein function or whether there is a formation of toxic proteins in vivo. In fact, several studies have shown that truncated ataxin-3 protein can cause normal cell dysfunction and neuronal damage by changing protein conformation, affecting mitochondrial function, interfering with protease hydrolysis system, and other aspects, resulting in neurotoxic effects [60–62]. Therefore, selective permanent inhibition and even precise repair of m*ATXN3* without affecting wt*ATXN3*, and achieving permanent gene silencing, is the ultimate targets of treatment. Previous studies have suggested that CRISPR/Cas9 genome editing based on HR can perform accurate gene repair for mutant genes [56], and pairs of sgRNAs designed for target genes can improve the cleavage efficiency of CRISPR/Cas9 genome editing [28]. Therefore, in this study, the CRISPR/Cas9 genome editing technique of precise gene repair based on HR and paired sgRNAs will be a promising treatment strategy in polyQ diseases.

The traditional Cre-loxP-mediated HR system we used is an operational tool for specific loci gene targeting [63]. In this study, HR-based CRISPR/Cas9 genome-editing technology was used to knock out and repair the abnormal CAG expansions, which was consistent with previous gene repair strategies for HD and SCA12 [10, 30, 64]. Unlike previous genome-editing studies of CRISPR/Cas9 in SCA3/MJD, paired sgRNAs and HR strategy are used for the first time to repair the abnormal CAG expansions in the exon 10 of *ATXN3* in SCA3-iPSCs, which can avoid the problems caused by the NHEJ repair induced truncated ataxin3. In addition, once we design and customize the sgRNAs targeted specific mutant expanded alleles, we should be able to implement expanded allele-specific editing by CRISPR/Cas9 genome therapy, such as SCA1. SCA2, SCA6, HD, etc. The above results confirmed that the genome-editing strategy used in this study can be used to knock out and repair target genes, with specificity and low OTs.

With reference to previous cerebral cortex neurons [40, 42] and cerebellum PCs [44–46, 65, 66] neural differentiation process. SCA3-iPSCs can differentiate into NESTIN and PAX6-positive NSCs and KIRREL2-positive Purkinje precursor cells. There was no significant difference in the expression of surface markers in NSCs derived from healthy controls iPSCs (Ctrl-iPSCs), SCA3-iPSCs, and corrected SCA3-iPSCs. In the mature cerebral cortical neurons, mixed neuronal cells expressing positive neuronal surface markers and positive astrocyte surface markers could be differentiated. There were no significant differences in the expression of neuronal cell surface markers and neuronal synaptic protein markers in neuronal cells derived from Ctrl-iPSCs, SCA3-iPSCs, and corrected SCA3-iPSCs. In the detection of electrophysiological function, mature cerebral cortical neurons could produce an action potential, whole-cell currents, and postsynaptic potential. no discernable differences were observed in EPSC amplitude and frequency, membrane capacitance, input resistance, and resting membrane potential among the groups. These results suggest that mt*ATXN3* does not affect the differentiation and electrophysiological function of NSCs stage and mature neurons, which largely reflected the heterogeneity of neuron types caused by the adopted differentiation protocols, rather than the differences caused by genotypic mutations. Consistent with previous HD studies, that did not affect neuronal differentiation maturity and electrophysiological parameters [10].

The present study also found that IC2-positive polyQ aggregations could be detected in SCA3 neuronal cells, which is a marker of neuronal degeneration and neuronal cell death [7, 24]. In polyQ disease stem cell models such as SCA3, previous studies have shown that polyQ aggregations and neurotoxic effects can only be detected under exogenous stressor stimulation or genetic manipulation [42, 67–69]. The afore-mentioned results confirmed the formation of the aggregates in SCA3 cell models under normal stress-free culture conditions, which may relate to its neurotoxicity, similar to previous studies [70, 71]. Moreover, In the absence of exogenous stressors, the human cell model showing endogenous expression of m*ATXN3* aggregations will be a good tool to help us further understand the role of endogenous *ATXN3* misfolding and aggregation in the pathogenesis of SCA3. However, the polyQ aggregations were not detected in the isogenic-controlled SCA3 neurons, suggesting that the disease phenotypes were improved, considering the mut*ATXN3* edited by CRISPR/Cas9, the mutant ataxin-3 protein could not occur in the iPSCs stage, and the aggregated could not form when differentiated into mature cortical neurons.

Studies have shown that mitochondrial dysfunction and oxidative stress disorder is involved in the pathogenesis of polyQ diseases, such as SCA3, and are also one of the research hotspots at present [48, 72, 73]. Studies have shown that abnormal aggregation of polyQ extended mutant ataxin3 protein can induce conformational changes of ataxin-3 protein, leading to misfolding and aggregation of the protein, which in turn affects mitochondrial function, leading to mitochondrial DNA damage, decreased MMP, and increased Ca^2+^ influx in SCA3 cell models and animal models [74–76]. In addition, studies have shown that abnormal aggregation of polyQ extended mutant ataxin-3 protein may lead to weakened binding with other antioxidant factors, resulting in the decline of GSH and other antioxidant enzymes, thereby activating oxidative stress response, leading to increased ROS production and triggering lipid peroxidation reaction. MDA production increases and eventually leads to neuronal damage in SCA3 cell models and animal models [77–79]. In this study, the polyQ extended mutant ataxin-3 protein may affect mitochondrial dysfunction and oxidative stress levels through the above mechanisms, while the decrease of MMP and GSH levels and the increase of Ca^2+^, ROS, and MDA were improved in corrected SCA3 neurons. These indicated that the abnormal phenotype of mitochondrial dysfunction and oxidative stress disorder was rescued in isogenic-controlled SCA3 neurons.

In the future, we will further explore the following aspects. Firstly, transcriptome changes in SCA3 and genetically controlled cell lines were performed to explore potential gene therapeutic targets. Secondly, cortical neurons differentiated by iPSCs cannot fully represent the neuropathological changes of SCA3 [11]. In addition, the decrease of PCs discharge frequency in SCAs mice was related to the abnormality of the K^+^ channel [23, 52], and electrophysiological function monitoring was needed after further differentiation to PCs in the future. Thirdly, m*ATXN3* may affect the mitochondrial respiratory function and energy metabolism disorder, which is related to the biological energy deficiency caused by mitochondrial dysfunction [3, 80]. However, the cellular bioenergy measurement has not been carried out in this study, and its relationship with cellular bioenergy and respiratory function should be further explored in future studies.

## Conclusions

In summary, this study proved for the first time that SCA3-iPSCs could be accurately repaired by using the paired sgRNAs/Cas9n and Cre-loxP-mediated HR strategy, and genetically repaired SCA3-iPSCs did not have the expression of the mutant ataxin-3 protein. In addition, we found that isogenic control SCA3-iPSCs retained pluripotent characteristics and normal karyotypes, and were able to differentiate into neuronal cells, and had electrophysiological characteristics. Besides, phenotypic abnormalities were identified in SCA3-NCs, including mitochondrial dysfunction and oxidative stress disorders, above disease phenotype, all were improved in corrected SCA3 neurons. Thus, this study accurately repaired SCA3-iPSCs based on CRISPR/Cas9 and HR strategies, providing a powerful and attractive gene therapy strategy for the future treatment of polyQ diseases, such as SCA3, and exploring fundamentally reverse the disease phenotype of polyQ diseases effectively in vitro experiments.

## Supplementary information

Supplementary materials
