# Supplementary material for: CRISPR/Cas9 mediated gene correction ameliorates abnormal phenotypes in spinocerebellar ataxia type 3 patient-derived induced pluripotent stem cells
Source: Transl Psychiatry. 2021 Sep 17;11:479. doi: 10.1038/s41398-021-01605-2 (PMC8448778; doi:10.1038/s41398-021-01605-2)
Supplement: Supplementary file 1 — Supplementary materials [file 41398_2021_1605_MOESM1_ESM.docx]

**Supplementary materials**


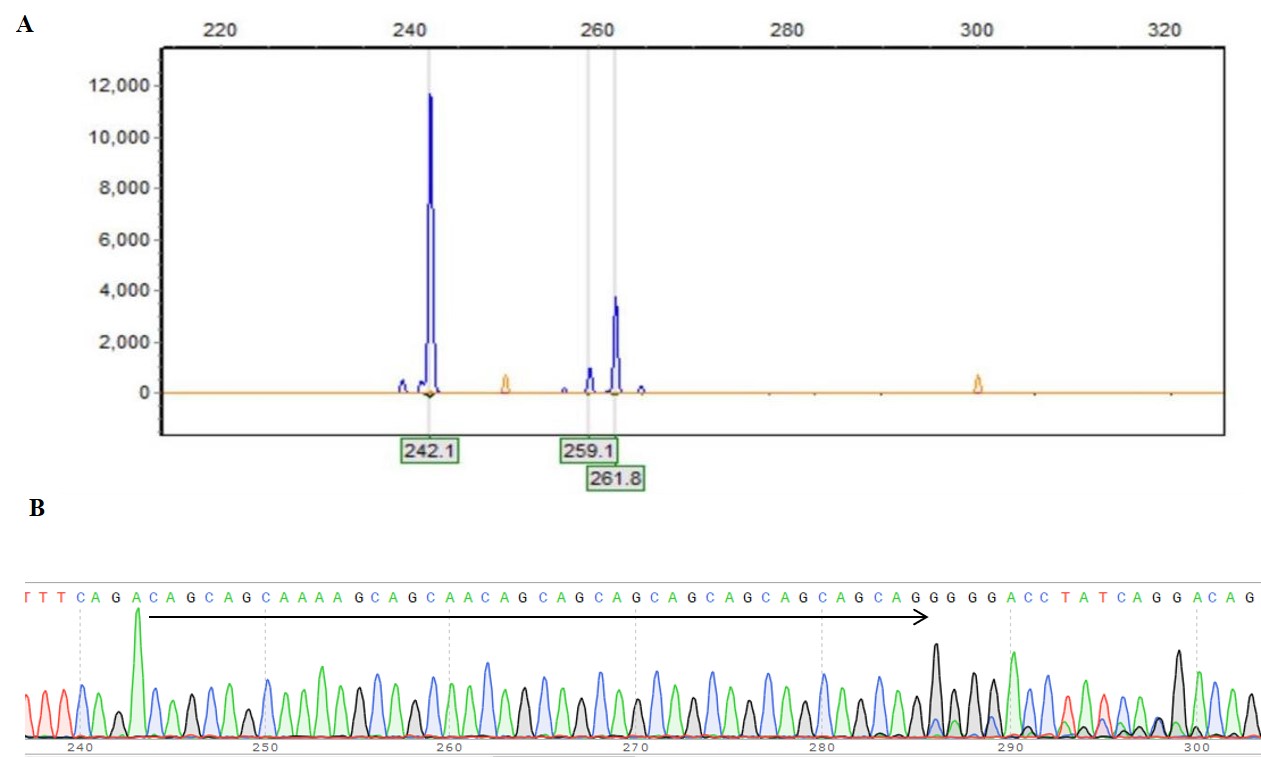


**Fig. S1 The CAG repeats of *ATXN3* in HEK293T cells.** The 14 and 21 CAG repeats of *ATXN3* was detected by capillary electrophoresis (**a**) and sequencing analysis (**b**).

**
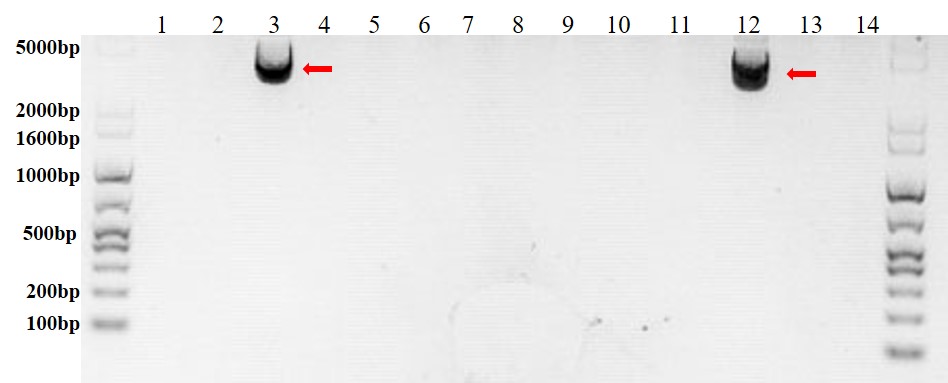
**

**Fig. S2 The positive clones were further verified by PCR verification.** C3 and C12 clones retained the targeted bands using P3~P4 primers.


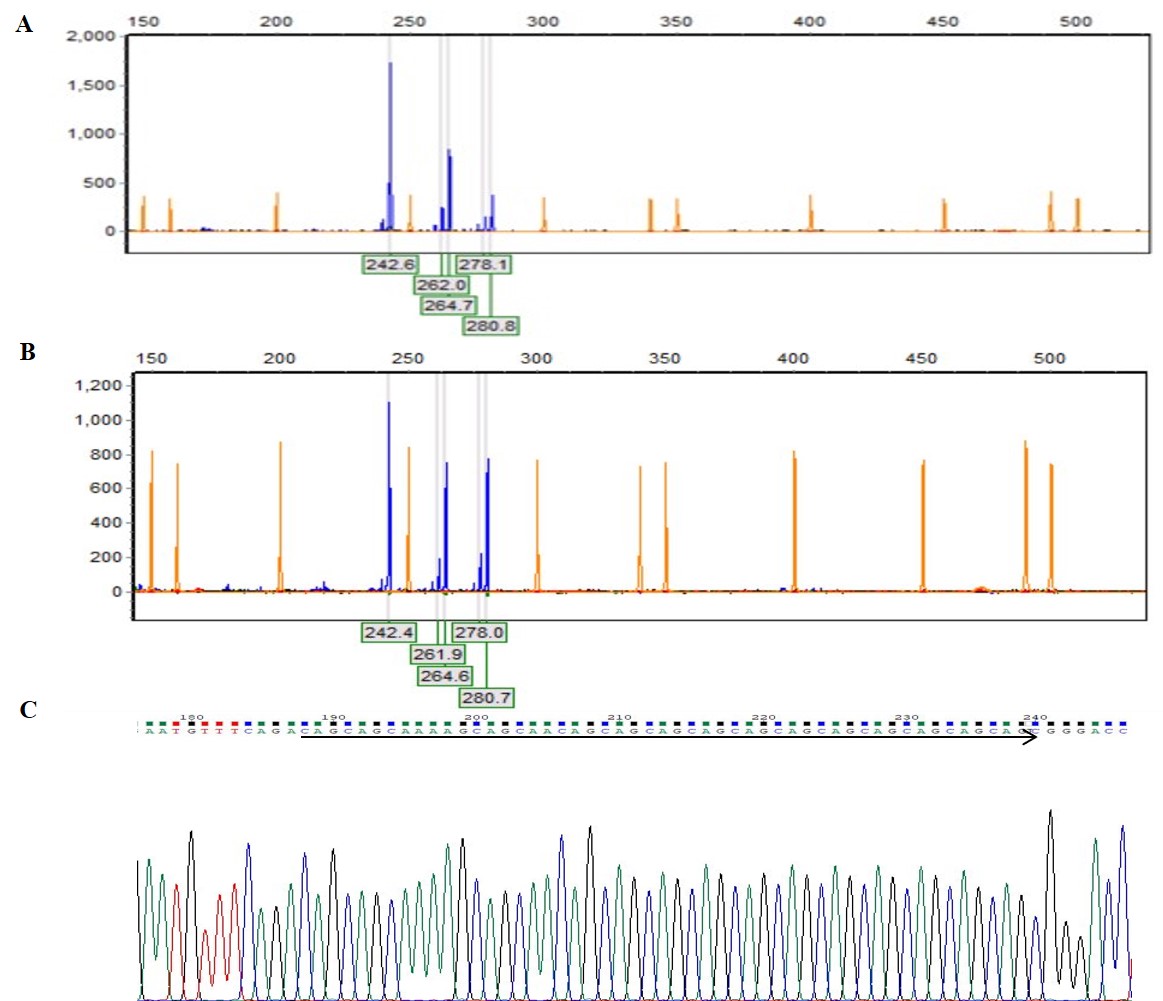


**Fig. S3 The CAG repeats of *ATXN3* in isogenic control SCA3-iPSCs were detected by capillary electrophoresis and Sanger sequencing analysis.** SCA3-C3 (**a**) and SCA3-C12 (**b**) isogenic control iPSCs indicated the peaks of 242.2 or 242.4 (17 CAG repeats) and 280.8 or 280.7 (31 CAG repeats), and Ta vector cloning the targeted bands by Sanger sequencing analysis showed the corrected cell lines contain 17 CAG repeats (**c**).

**
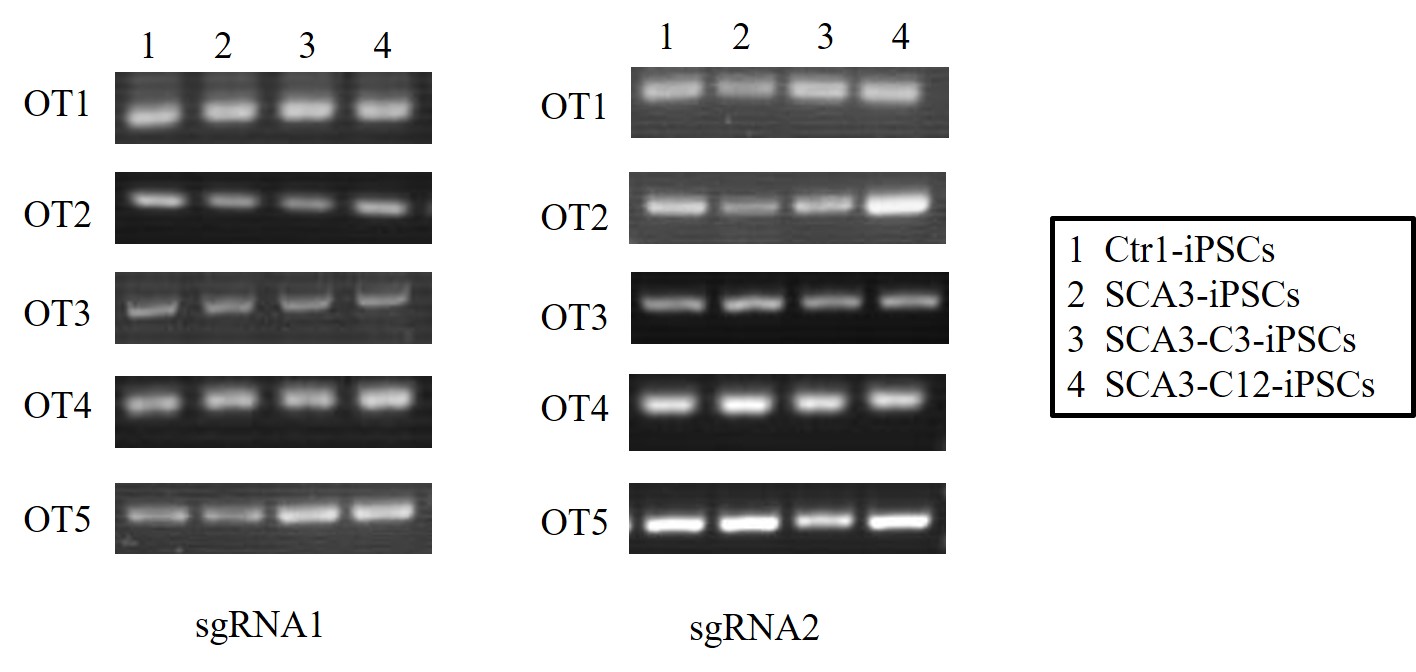
**

**Fig. S4 The predicted off-targeted locis were verified by T7EN1 assay.** sgRNA1 and sgRNA2 were analyzed by T7EN1 assay, the results didn’t detect any potential off-targeted sites.


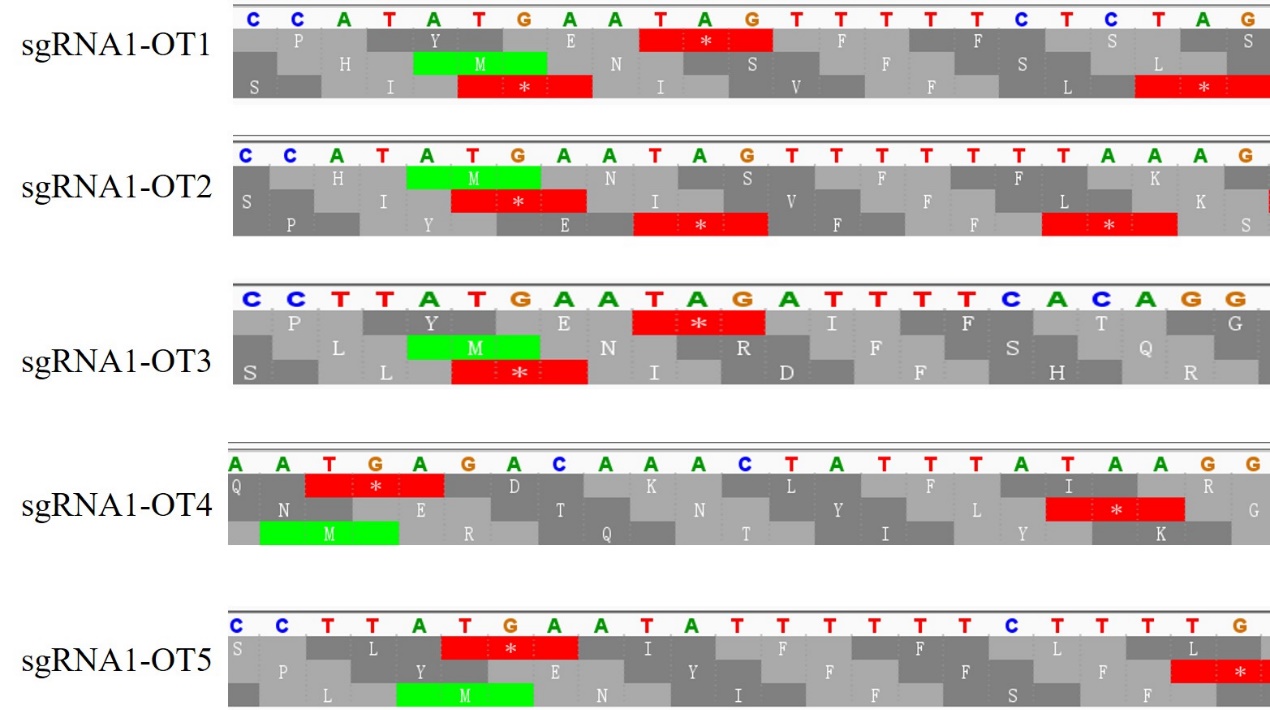


**Fig. S5 Whole genome sequencing analysis of off target sgRNA1.** No potential off target loci of sgRNA1 were detect by whole genome sequencing


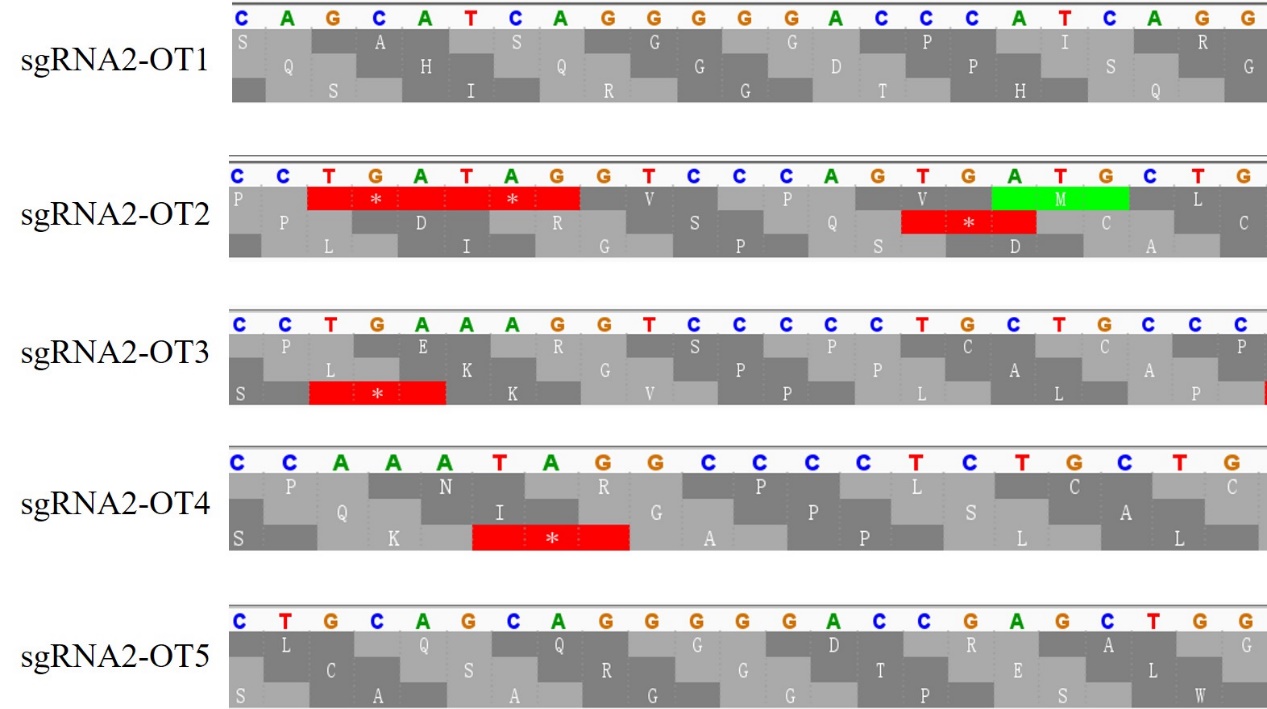
**Fig. S6 Whole genome sequencing analysis of off target sgRNA2.** No potential off target loci of sgRNA2 were detect by whole genome sequencing


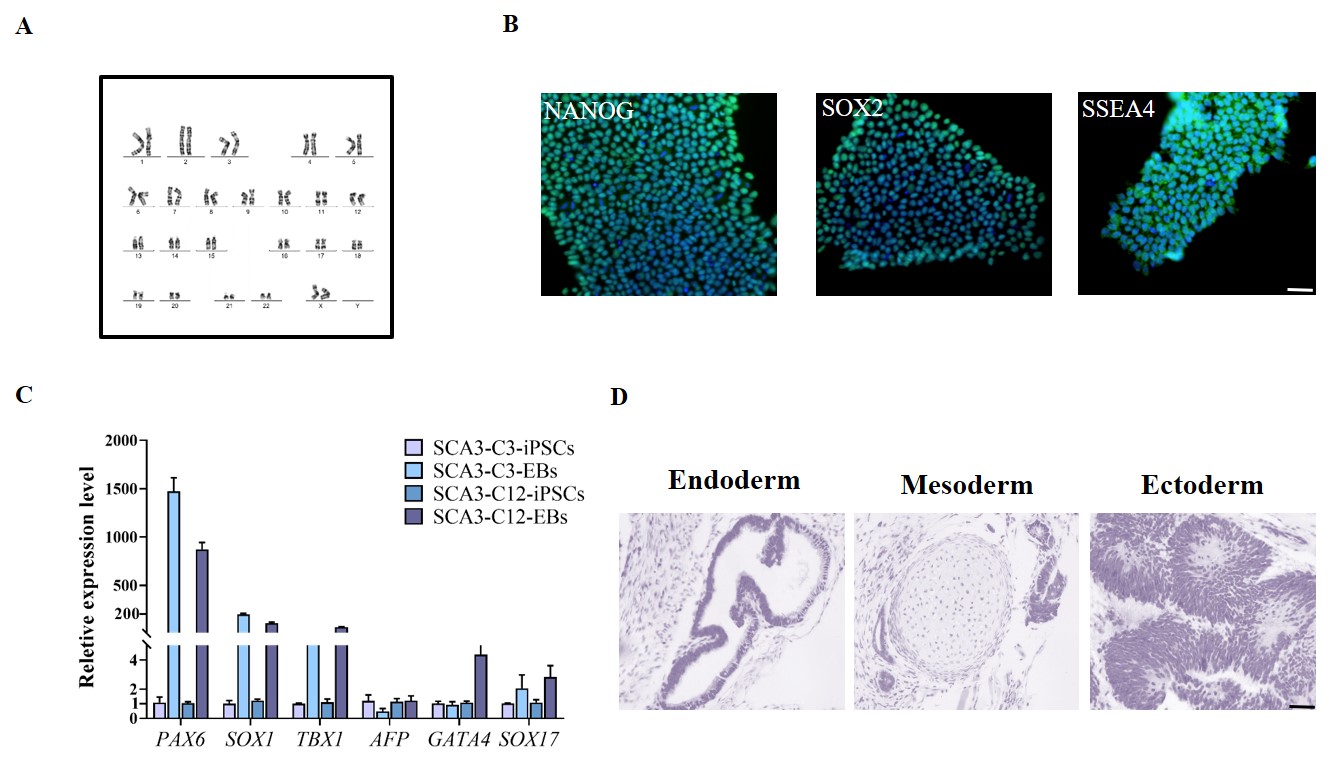


**Fig. S7 The characterization of corrected SCA3-iPSCs (SCA3-C12-iPSCs).** **a** karyotype analysis showed the corrected SCA3-C12-iPSCs have a normal 46, XX karyotype. **b** Immunofluorescence analysis of pluripotent markers, such as NANOG, SOX2 and SSEA4. Scale bar: 100μm. **c** RT-qPCR analysis of human embryonic stem cell markers for the three germ layers relative to iPSCs. The SCA3-C12-iPSCs had the potential to differentiate into three dermal layers. n=3 in every independent biological replicates sample. **d** Teratomas test suggested the iPSCs could differentiate into 3 germ layers. Scale bar: 100µm.


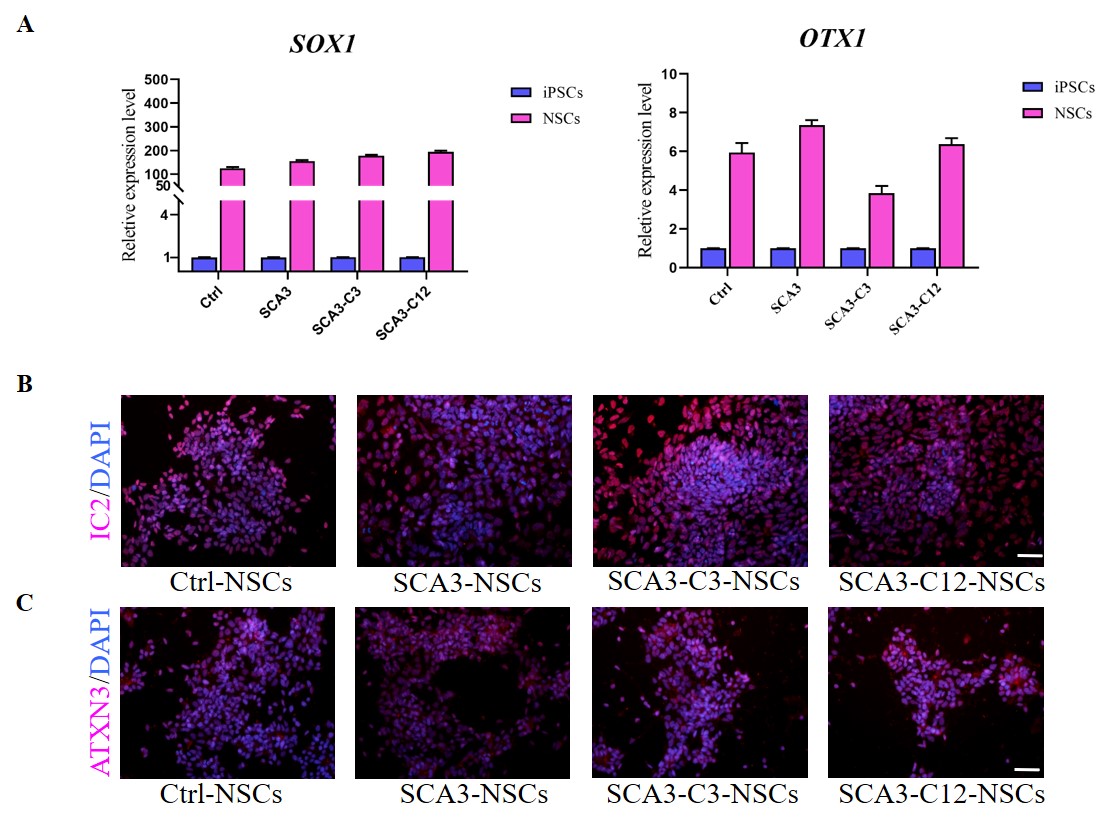


**Fig. S8 The NSCs expression marker and polyQ aggregates detected by RT-qPCR and immunofluorescence. a** The NSCs markers of *SOX1*, and *OTX1* (day 16) were analyzed by RT-qPCR. The differentiation at days 16~20 indicated the NSCs markers significantly increased compared to undifferentiated iPSCs. n=3 in every independent biological replicates sample. **b-c** Ctr1-NSCs, SCA3-NSCs and isogenic SCA3-NSCs were stained for ATXN3 and IC2, no polyQ aggregates were detected in each group, Scale bar: 100µm. Ctrl-NSCs: healthy control NSCs. SCA3-NSCs: SCA3 patient derived NSCs. SCA3-C3-NSCs and SCA3-C12-NSCs: corrected SCA3-NSCs.


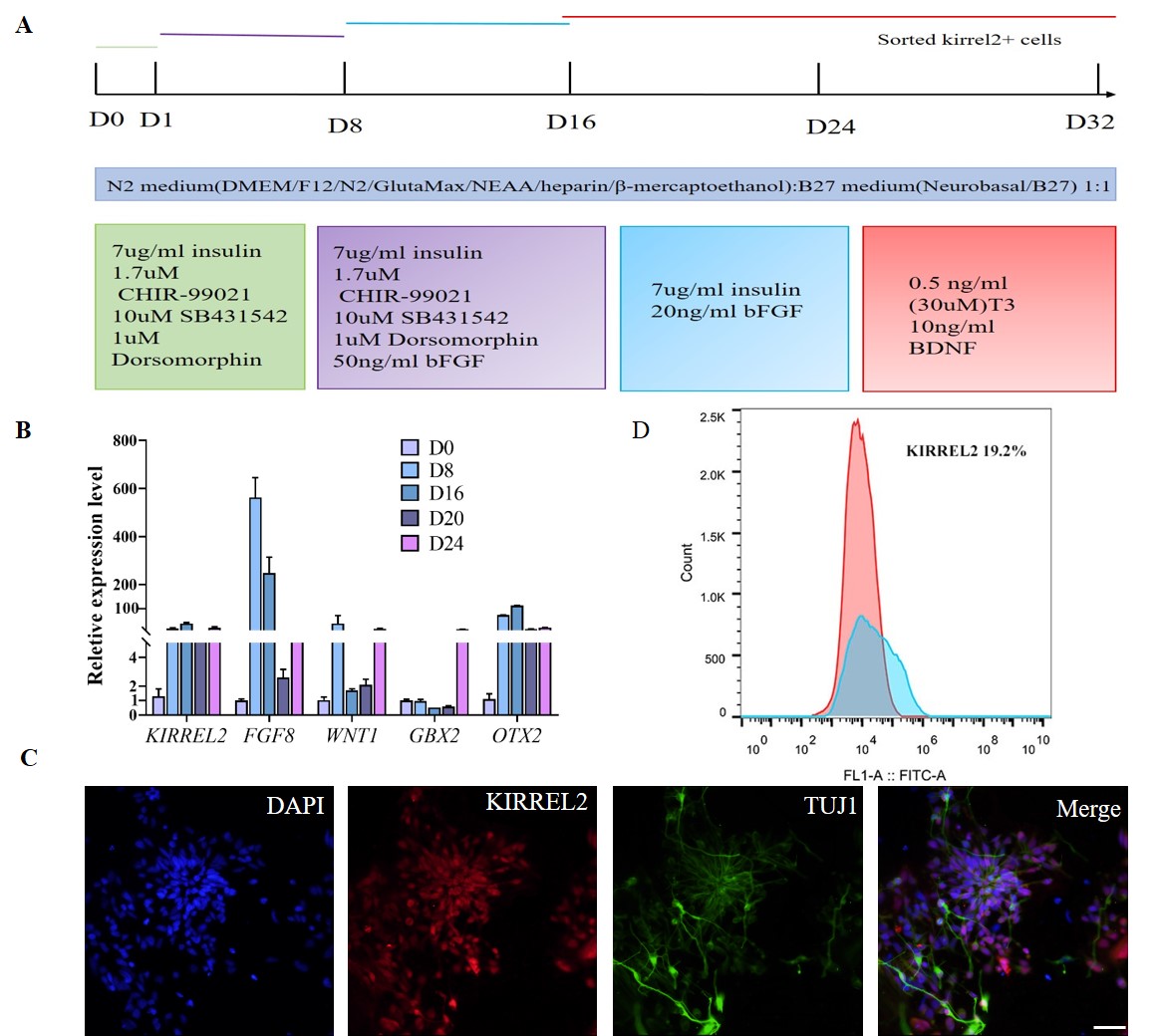


**Fig. S9 Characterization of Purkinje precursor cells from SCA3-iPSCs.** a Schematic representation of the in vitro Purkinje precursor cells differentiation protocol. **b** Quantitative RT-qPCR analysis of *KIRRL2*, *FGF8*, *WNT1*, *GBX2* and *OTX2* during the initial cerebellar patterning of iPSCs at days 0~24 of differentiation, SCA3 Purkinje precursor cells compared with undifferentiated hiPSCs (fold change = 1). **c** Mature Purkinje cells progenitor markers KIRREL2/TUJ1 at days 24~32 of differentiation detected by immunofluorescence. Scale bar: 100μm. **d** Representative images of flow cytometry analysis at day 32 of SCA3-iPSC derived cerebellar Purkinje precursor cells populations were 19.2%.


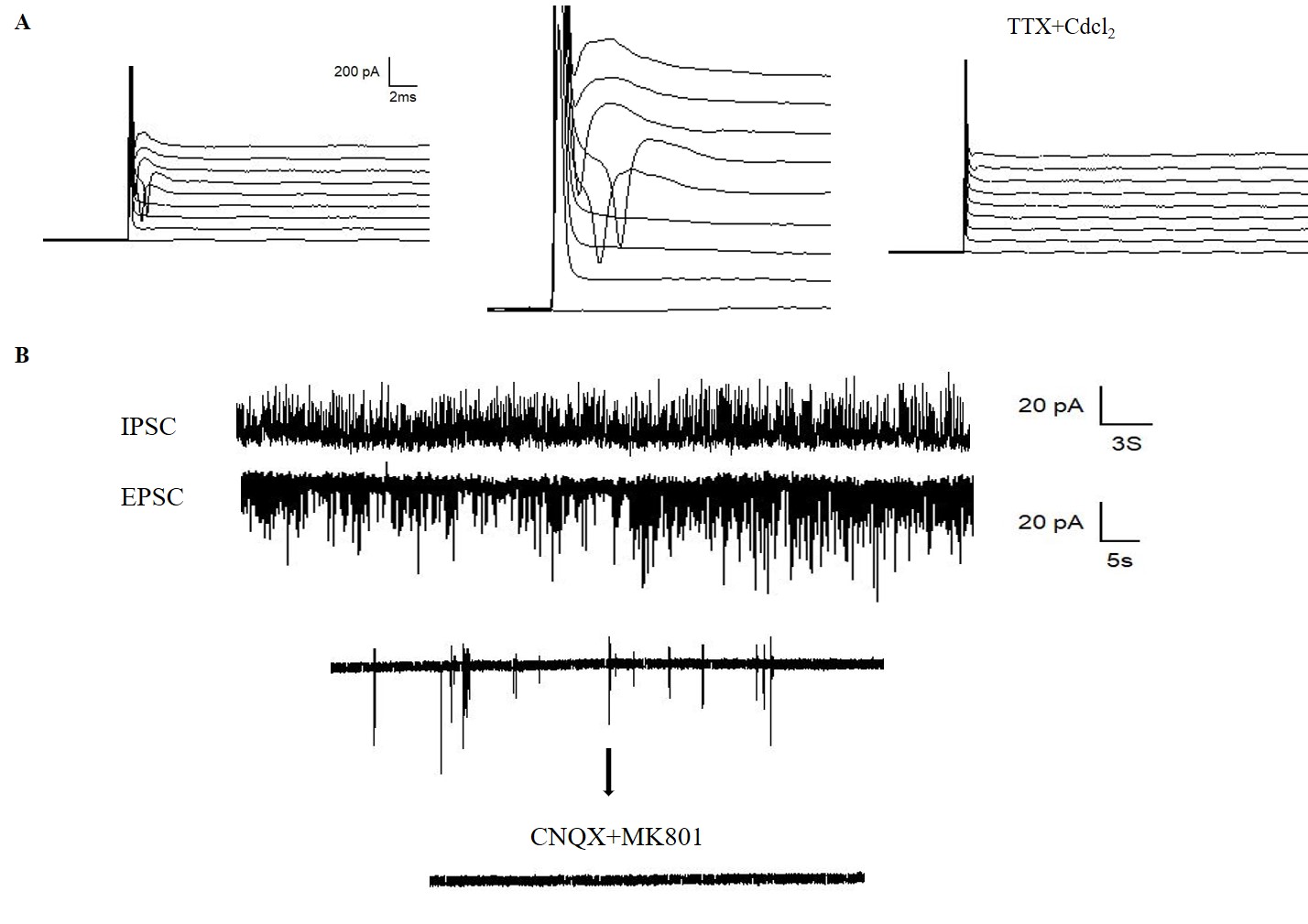


**Fig. S10** Characterization of electrophysiological function from SCA3/MJD-iPSCs **a** The inward (Na^+^ and Ca^+^) and outward currents (K^+^) were blocked by TTX (1μM) and Cdcl_2_ (0.1mM) ion antagonist, respectively. **b** Representative traces of inhibitory postsynaptic current (IPSC, upper image) and excitatory postsynaptic current (EPSC, bottom image), the EPSC were blocked by CNQX and MK801.


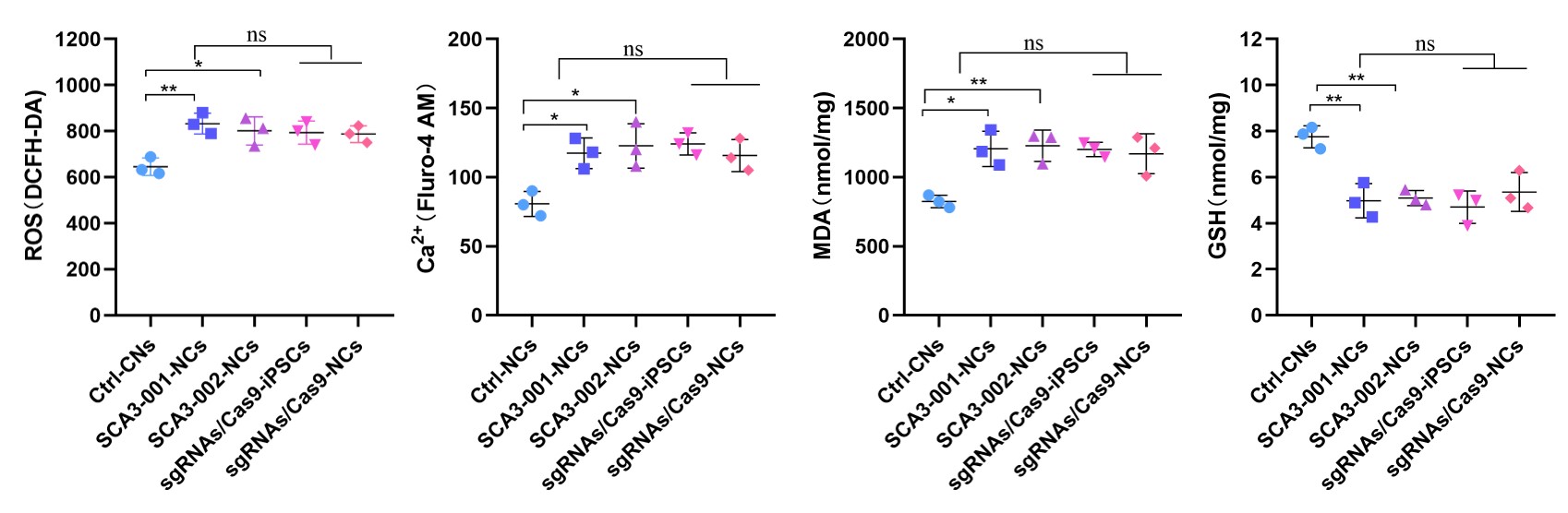


**Fig. S11** The acute treatment of sgRNAs/Cas9 of SCA3-iPSCs derived neurons **a-d** There were no significant difference among the SCA3-NCs（SCA3-001-NCs and SCA3-002-NCs）, sgRNAs/Cas9-iPSCs and sgRNAs/Cas9-NCs. Ctrl-NCs: healthy control neurons, SCA3-NCs (SCA3-001-NCs and SCA3-002-NCs): SCA3-001-iPSCs patient derived iPSCs and SCA3-001-iPSCs patient derived iPSCs, sgRNAs/Cas9-iPSCs: acute treatment sgRNAs/Cas9 into iPSCs, sgRNAs/Cas9-NCs: cute treatment sgRNAs/Cas9 into neurons. Data were showed as mean ± SD (n=3). The data as calculated using one-way ANOVA, followed by Bonferroni post-hoc test. *P<0.05 ,**P<0.01, ***P<0.005, ****P<0.001.

Table S1 The designed sequences of *ATXN3*-sgRNA1 and *ATXN3*-sgRNA2

| Name |  | Sequence |
| --- | --- | --- |
| *ATXN3*-sgRNA1 | Forward | 5’-CACCGCATGAGAAAAACTATTCATA-3’ |
|  | Reverse | 5’-AAACTATGAATAGTTTTTTCTCATGC-3’ |
| *ATXN3*-sgRNA2 | Forward | 5’-CACCGCAGCAGCAGGGGGACCTATC-3’ |
|  | Reverse | 5’-AAACGATAGGTCCCCCTGCTGCTGC-3’ |

Table S2 Primers for PCR

| Gene | Forward Primer (5’-3’) | Reverse Primer (5’-3’) |
| --- | --- | --- |
| *ATXN3*-FAM | CCAGTGACTACTTTGATTCG-FAM | CTTACCTAGATCACTCCCAA |
| P1~P2 | AGCACTTCCATATTTTAAAGTAATCTG | TGCTCCTTAATCCAGGGAAA |
| P3~P4 | AGTGGGAGTGGATGCTGAGT | ATGCTGCCCATAAGGTATCG |
| P5~P6 | AGATTTCCTAAGATCAGCACTTCC | TGGAGACTGTACAAATTACTGTGA |

Table S3 The potential off-targeted locis of sgRNA1 and sgRNA2

| Predicted OTs | Sequence | Location |
| --- | --- | --- |
| SgRNA1 |  |  |
| OT-1 | C**TA**GAGAAAAACTATTCATA**T**GG | Chr10: 65064883-65064905 |
| OT-2 | C**T**T**T**A**A**AAAAACTATTCATA**T**GG | Chr 2: 57984217-57984239 |
| OT-3 | C**C**TG**T**GAAAA**T**CTATTCATA**A**GG | Chr17: 5961399-5961421 |
| OT-4 | **A**ATGAGA**C**AAACTATT**T**ATA**A**GG | Chr8: 88653942-88653964 |
| OT-5 | CA**AA**AGAAAAA**A**TATTCATA**A**GG | Chr3:165679124-165679146 |
| SgRNA2 |  |  |
| OT-1 | CAGCA**T**CAGGGGGACC**C**ATC**A**GG | Chr7:128847638-128847660 |
| OT-2 | CAGCA**T**CA**CT**GGGACCTATC**A**GG | Chr7: 30199832-30199854 |
| OT-3 | **GG**GCAGCAGGGGGACC**T**TTC**A**GG | Chr 8: 65765130-65765152 |
| OT-4 | CAGCAGCAG**A**GGG**G**CCTAT**TT**GG | Chr20: 4295014-4295036 |
| OT-5 | C**T**GCAGCAGGGGGACC**G**A**G**C**T**GG | Chr20: 44919303-44919325 |

Table S4 Primers for RT-qPCR

| Gene | Forward Primer (5’-3’) | Reverse Primer (5’-3’) |
| --- | --- | --- |
| *NANOG* | TGAACCTCAGCTACAAACAG | TGGTGGTAGGAAGAGTAAAG |
| *SOX2* | CCCAGCAGACTTCACATGT | CCTCCCATTTCCCTCGTTTT |
| *OCT4* | CCTCACTTCACTGCACTGTA | CAGGTTTTCTTTCCCTAGCT |
| *PAX6* | TTGCTTGGGAAATCCGAG | GGAGCCTGAGCTTCTTAGCA |
| *NESTIN* | CAGGGGCAGACATCATTGGT | CAGGGGCAGACATCATTGGT |
| *FOXG1* | AGGAGGGCGAGAAGAAGAAC | TCACGAAGCACTTGTTGAGG |
| *OTX1* | GCCTCCCCTTCCAGTCTTTC | GGGCAGAAACACGCCAGTTA |
| *SOX1* | TACAGCCCCATCTCCAACTC | GCTCCGACTTCACCAGAGAG |
| *GAPDH* | ACACCCACTCCTCCACCTTT | TTACTCCTTGGAGGCCATGT |
| *FGF8* | GAGCCTGGTGACGGATCAG | CGTTGCTCTTGGCGATCAG |
| *WNT1* | CAACCGAGGCTGTCGAGAAA | GTGCAGGATTCGATGGAACCT |
| *KIRREL2* | GGGGCTAGTTCAGTGGACTAA | CACGGGCCTAATGTGGAGG |
| *GBX2* | GACGAGTCAAAGGTGGAAGAC | GATTGTCATCCGAGCTGTAGTC |
| *OTX2* | AGAGGACGACGTTCACTCG | TCGGGCAAGTTGATTTTCAGT |

Table S5 Primary and secondary antibodies

| Antibody | Company | Host Species | Catalog number | Dilution |
| --- | --- | --- | --- | --- |
| Primary Antibodies | | | | |
| ATXN3/ataxin3 | Millipore | Mouse | MAB5360 | 1: 500 |
| PolyQ-IC2 | Millipore | Mouse | MAB1574 | 1:1000 |
| NANOG | Cell Signaling | Rabbit | D73G4 | 1:200 |
| SOX2 | Cell Signaling | Rabbit | D3579 | 1:400 |
| SSEA4 | Bioss | Rabbit | Bs309R | 1:200 |
| PAX6 | Abcam | Rabbit | Ab5790 | 1:50 |
| NESTIN | Abcam | Chicken | Ab134017 | 1:10000 |
| KIRRLE2 | Sigma | Mouse | WH0084063M1 | 1:100 |
| TUJ1 | Abcam | Rabbit | Ab195879 | 1:400 |
| MAP2 | Abcam | Rabbit | Ab5622 | 1:200 |
| GABA | Abcam | Mouse | Ab0310 | 1:100 |
| GFAP | Abcam | Mouse | Ab33922 | 1:400 |
| Synapsin-1 | Cell Signaling | Rabbit | D12G5 | 1:200 |
| PSD-95 | Invitrogen | Mouse | 6G6-1C9 | 1:500 |
| GADPH | Bioworld | Rabbit | MB001H | 1:5000 |
| DAPI | Beyotime | - | C1002 | 1:10000 |
| Secondary Antibodies | | | | |
| Anti-Rabbit -488 | Proteintech | Goat | SA00006–2 | 1:400 |
| Anti-Mouse- 594 | Proteintech | Goat | SA00006–3 | 1:400 |
| Anti-Mouse-488 | Invitrogen | Donkey | A32766 | 1:400 |
| Anti-Chicken-594 | Jackson | Chicken | 703-585-155 | 1:400 |
| Anti-Rabbit -594 | Invitrogen | Donkey | R37119 | 1:400 |
| HRP-Rabbit | Beyotime | - | A0208 | 1:1000 |
| HRP- Mouse | Beyotime | - | A0216 | 1:1000 |

Table S6 Summary of capture statistics for whole genome sequencing

| Samples | Raw reads | Clean reads | Clean data rate（%） | Clean read Q20（%） | Clean read Q30（%） | GC content（%） |
| --- | --- | --- | --- | --- | --- | --- |
| SCA3-iPSCs-001 | 673,964,372 | 666,112,780 | 99.84 | 97.81 | 94.07 | 40.58 |
| SCA3-C3-iPSCs | 674,012,242 | 666,025,066 | 98.81 | 95.77 | 90.05 | 40.30 |
| SCA3-C12-iPSCs | 672,400,214 | 666,078,202 | 99.06 | 95.90 | 90.23 | 41.72 |

**Supplemental** **Experimental Procedures**

**Genomic PCR, and repeat length analysis**

Genomic DNA was extracted using the genomic DNA kit (TIANGEN, China). The CAG repeats in the 10 exon of the *ATXN3* was amplified by PCR with ﬂuorescent primer (Table S2), and the repeat length analysis was carried out by capillary electrophoresis on ABI PRISM 3130 Genetic analyzer (Applied Biosystems, China). Short tandem repeat (STR) analysis was analysis by GeneMarker software 4.0. The expanded *ATXN3* band can be obtained through DNA gel extraction kit (TIANGEN, China) and pMDTM18-Ta cloning. Sanger sequencing of the CAG repeats was carried out in Beijing TsingKe Biotechnology Company. The sequencing was analyzed by Chromas software

**Whole genome sequencing**

1 μg high molecular weight DNA per sample was used for whole genome sequencing. DNA splice was performed using the Covaris M220 focused ultrasound (Covaris Inc, USA) with an average fragment size of 350bp. The cleavage was followed by 3’ terminal repair, purification, and PCR product cyclization. Before genome capture, the DNA concentration was detected, and the qualified libraries were sequenced (DNBSEQ). The SCA3-C3, SCA3-C12 and CAG74 samples were sequenced by 101.1 Gb, 100.8 Gb, and 101.1 Gb, respectively, with average read lengths of 33.83×, 33.90× and 34.00×, respectively. The average coverage of each base in the genome group was 98.96% (SCA3-C3), 98.96% (SCA3-C12) and 98.97% (CAG74), respectively, and 92.52% of the bases covered at least 20×. The human reference genome [Human GRCH37 (HG19) build] was read using the Burrows-Wheeler Aligner （BWA）. PCR duplicates in the BAM file were recognized and deleted by the Samtools (v1.3.1). BAM file were runned by Haplotypecaller and CNVnator (v0.3.2 ) of GATK (v4.1.4.1) software. Singlenucleotide polymorphism (SNP), copy number variation (CNV) and potential sgRNAs off target loci were analyzed.

**Characterization of corrected SCA3-iPSCs**

The corrected SCA3-iPSCs were identified for normal karyotype and pluripotency characterization, including karyotype analysis, immunofluorescence, flow analysis and RT-qPCR detection for pluripotency gene expression, differentiation potential in vivo were detected by teratoma assay, these experiments were proceeded according to the previous protocols^1-3^.

**Purkinje progenitor cells differentiation**

According to the Purkinje cells (PCs) differentiation protocols^4-7^. iPSCs were dissociated into single cells using accutase for 5 minutes at 37 °C. Single cells were plated into low-attachment dishes (CLS3471, Corning, USA) to form aggregates in neural induction medium: DMEM/F12 and Neurobasal medium in the mix ratio of 1:1, supplement with 1×N2, 1×B27, 1% NEAA, 1% GlutaMax (all from Life Technologies, USA) , 100µM beta-mercaptoethanol (Sigma, USA), 2ug/mL heparin (Sigma, USA), 10 µM SB-431542 (Selleck, USA), 1 µM Dorsomorphin (Sigma, USA), 1.7µM CHIR99021 (Selleck, USA) , 10 µM Y-27632 (Selleck, USA) and 7ug/mL insulin (sigma, USA). At days 2 of differentiation, 50ng/mL bFGF (PeproTech, USA) was added to the medium. Cells were plated into PDL (50 µg/mL) and laminin (10µg/mL) coated plates at days 8, and SB431542, Dorsomorphin, CHIR99021 and Y-27632 were removed at days 8. At days 16 the neural rosette were occurred, 10 ng/mL BDNF (PeproTech, USA), 30 µM T3 (Sigma, USA) were added in the medium for Purkinje precursor cells expansion, the purified Purkinje precursor cells through KIRREL2-selection by Flow cytometry (FACS) at days 24~32 of differentiation.

**Western blot analysis**

Protein samples were collected into RIPA-buffer supplemented with PMSF 2mM, protease inhibitor cocktail (Beyotime, China). Protein concentration was analyzed using BCA protein concentration determination kit (Beyotime, China). Cell lysates were subjected to 10~12% polyacrylamide gel electrophoresis (PAGE) for 2h, the nitrocellulose membrane was added to the transfer buffer and iced-transferred at a constant voltage (100V) for 1 hour. Incubate primary antibody 4℃ through the night. Western blot antibody with ataxin-3 (MAB5360, Millipore, USA). The secondary antibody was incubated at room temperature, and imprinting was performed with ECL electrochemiluminescence detection kit (Thermo Fisher Scientific, USA). Signals were carried out by the WB automatic MiniChemi™ chemiluminescence imaging system (Beijing Saizhi Venture Technology Company, China). Semi-quantitative analysis with Image J and GraphPad Prism 8.

**Flow cytometry**

Cells were detached from plates as single cells using accutase (Sigma, USA), and fixed in 1% paraformaldehyde for 30 min at 37 °C. The cells were resuspended in PBS contains 10% BSA and resuspended in 90% formaldehyde for permeabilization for 30 minutes at 4 °C. Primary antibodies were added for the cells and incubated for 30 minutes at RT (Table S5). After washes with 10% BSA-PBS, the secondary antibodies were added to the cells and incubated for 30 min at RT in dark. After the washes, cells were resuspended into 200~300μL BSA-PBS and filtered on ice until performed on the BD FACS Aria II (BD Biosciences, USA), the date analysis using the Flow Jo Software.

**ROS, Ca^2+^, MDA and GSH measurement**

ROS detection: 10μM DCFH-DA fluorescent probe (Beyotime, China) was added into the cell fluid. The final concentration was 10μm/L, incubated in an incubator at 37 ℃ for 20~30 min, washed with PBS three times, and removed DCFH-DA. The excitation wavelength of 488nm and the emission wavelength of 525nm were detected by LB943-Multifunctional reader (Berthold, Germany). H_2_O_2_-induced ROS detection: DCFH-DA fluorescent probe was used in the Reactive Oxygen Species Assay Kit (Beyotime, China) to detect the reactive oxygen levels in cells. After iPSCs were digested by 0.5% EDTA, 2×10^4^ cells were harvested and cultured in a 96-well plate for 48h for ROS examination. The cells were washed with PBS for 2~3 times. After incubation with a 10μm DCFH-DA fluorescent probe for 30min, the medium was discarded and cells were stimulated by adding 0.3% H_2_O_2_. ROS of each group cell (Ctrl-NCs, SCA3-NCs and isogenic control SCA3-NCs) were recorded on LB943-Multifunctional reader every 10min for a total of 10 times, data were plotted and analyzed using GraphPad Prism 8 software. Intracellular Ca^2+^ detection: Fluo-4 AM (Beyotime, China) for detecting intracellular Ca^2+^ concentrations, the cells were incubated with Fluo-4 AM at a final concentration of 1.5μM diluted in DMSO for 30 minutes, Then, the cell fluorescence intensity was detected at the excitation wavelength of 488 nm and emission wavelength of 516 nm. MDA and GSH detection: The cell lysates treated by RIPA were collected. Then, the supernatant was collected and analyzed, the Lipid Peroxidation Malondialdehyde (MDA) Assay Kit (Beyotime, China) and reduced glutathione/oxidized glutathione (GSH and GSSG Assay Kit, Beyotime, China) were measured according to the manufacturer's instructions.

**Reference**

1. He L, et al. Generation of an induced pluripotent stem cell line (XHCSUi001-A) from urine cells of a patient with spinocerebellar ataxia type 3. *Stem Cell Res* **40**, 101555(2019).

2. He L, et al. Generation of induced pluripotent stem cell line (CSUXHi002-A) from a patient with spinocerebellar ataxia type 1. *Stem Cell Res* **45**, 101816(2020).

3. He L, et al. Generation of spinocerebellar ataxia type 3 patient-derived induced pluripotent stem cell line (CSUXHi005-A) from human urine epithelial cells. *Stem Cell Res* **53**, 102289(2021).

4. Sundberg M, et al. Purkinje cells derived from TSC patients display hypoexcitability and synaptic deficits associated with reduced FMRP levels and reversed by rapamycin. *Mol Psychiatry* **23**, 2167-2183(2018).

5. Wang S, et al. Differentiation of human induced pluripotent stem cells to mature functional Purkinje neurons. *Sci Rep* **5**, 9232(2015).

6. Muguruma K, et al. Ontogeny-recapitulating generation and tissue integration of ES cell-derived Purkinje cells. *Nat Neurosci* **13**, 1171-1180(2010).

7. Muguruma K, Nishiyama A, Kawakami H, Hashimoto K, Sasai Y Self-organization of polarized cerebellar tissue in 3D culture of human pluripotent stem cells. *Cell Rep* **10**, 537-550(2015).
